# Efficacy of cannabinoids in neurodevelopmental and neuropsychiatric disorders among children and adolescents: a systematic review

**DOI:** 10.1007/s00787-023-02169-w

**Published:** 2023-03-03

**Authors:** Lauren J. Rice, Lisa Cannon, Navin Dadlani, Melissa Mei Yin Cheung, Stewart L. Einfeld, Daryl Efron, David R. Dossetor, Elizabeth J. Elliott

**Affiliations:** 1 The University of Sydney, Faculty of Medicine and Health, Specialty of Child and Adolescent Health, Sydney, NSW Australia; 2 Sydney Children’s Hospitals Network, Kids Research, Sydney, Australia; 3 Telethon Kids Institute, Perth Children’s Hospital, Perth, WA Australia; 4 The University of Sydney, Faculty of Medicine and Health, Brain and Mind Centre, Sydney, NSW Australia; 5 Department of General Paediatrics, Health Services, Murdoch Children’s Research Institute, Royal Children’s Hospital, Parkville, Australia; 6 Department of Paediatrics, University of Melbourne, Melbourne, Australia

**Keywords:** Cannabinoids, Neurodevelopmental disorder, Developmental disability, Paediatrics, Neuropsychiatric disorders

## Abstract

A better understanding of the endocannabinoid system and a relaxation in regulatory control of cannabis globally has increased interest in the medicinal use of cannabinoid-based products (CBP). We provide a systematic review of the rationale and current clinical trial evidence for CBP in the treatment of neuropsychiatric and neurodevelopmental disorders in children and adolescents. A systematic search of MEDLINE, Embase, PsycINFO, and the Cochrane Central Register of Trials was performed to identify articles published after 1980 about CBP for medical purposes in individuals aged 18 years or younger with selected neuropsychiatric or neurodevelopmental conditions. Risk of bias and quality of evidence was assessed for each article. Of 4466 articles screened, 18 were eligible for inclusion, addressing eight conditions (anxiety disorders (*n* = 1); autism spectrum disorder (*n* = 5); foetal alcohol spectrum disorder (*n* = 1); fragile X syndrome (*n* = 2); intellectual disability (*n* = 1); mood disorders (*n* = 2); post-traumatic stress disorder (*n* = 3); and Tourette syndrome (*n* = 3)). Only one randomised controlled trial (RCT) was identified. The remaining seventeen articles included one open-label trial, three uncontrolled before-and-after trials, two case series and 11 case reports, thus the risk of bias was high. Despite growing community and scientific interest, our systematic review identified limited and generally poor-quality evidence for the efficacy of CBP in neuropsychiatric and neurodevelopmental disorders in children and adolescents. Large rigorous RCTs are required to inform clinical care. In the meantime, clinicians must balance patient expectations with the limited evidence available.

**Supplementary Information:**

The online version contains supplementary material available at 10.1007/s00787-023-02169-w.

## Introduction

Interest in the potential therapeutic benefits of cannabis grew during the 1990s with increased understanding of the endocannabinoid system (ECS) in the brain [1]. The ECS plays an important role in the developing nervous system: it mediates crosstalk between neurotransmitter systems and regulates psychiatric, neurological, cardiovascular, endocrine and immunological functions [2, 3]. The ECS also helps regulate cognition, mood, and sleep [4–6]. Components of the ECS include the G-protein-coupled receptors cannabinoid receptor types 1 and 2 (CB_1_ and CB_2_); lipid-based retrograde endocannabinoid neurotransmitters; and enzymes that synthesise and degrade the endocannabinoids (endogenous cannabinoids). The CB_1_ receptors are abundant and widespread throughout the central nervous system with lower concentrations in the peripheral tissues and cells. CB_2_ receptors are mainly found on immune cells in the central nervous, immune, and haematopoietic systems as well as the pancreas and bones. Endocannabinoids are agonists of CB_1_ and CB_2_. The two most widely studied endocannabinoids are anandamide/*N*-arachidonoylethanolamide (AEA) and 2-Arachidonoylglycerol (2-AG) [7]. When evaluating the effects of ECS-targeting drugs it is important to consider that the ECS is not a discrete, isolated system. Rather, most components are multifunctional and interact with many other signalling pathways [8]. For a detailed description of the ECS, see Brown et al. [9].

In the early twenty-first century, growing social interest and evidence from preclinical (animal) studies for the therapeutic benefits of cannabinoid-based products (CBP) led to a global trend to relax regulatory restrictions [10]. Internationally, the legalisation of cannabis is rapidly changing both as a recreational and medicinal product [11]. Recreational cannabis use has been legalised in Canada, Georgia, Malta, Mexico, South Africa, Thailand, Uruguay, nineteen states in the United States (US) of America and one state in Australia. Medicinal cannabis use has been legalised in 45 countries and 37 US states [12].

The Australian Department of Health expected 70,000 prescriptions for medical cannabis to be filled in Australia by the end of 2020 [13]. By August 2021, 5288 approvals to access medical cannabis for category B (non-seriously ill) patients had been granted for children aged 2–18 years. Interestingly, the most common indications approved were for autism spectrum disorder (ASD; 1580 approvals) and anxiety disorder (1260 approvals), which both had more approvals than epilepsy and seizure disorders combined (1128) despite there being more preliminary evidence for some and seizure disorders [14, 15]. There were also 267 approvals for attention deficit hyperactivity disorder (ADHD) and 55 for challenging behaviour. [16]. These data highlight the growing interest in medicinal cannabis for paediatric neurodevelopmental and neuropsychiatric disorders. Psychiatrists are the third most frequent prescribers of medical cannabis in Australia after general practitioners and neurologists [13] and there is increasing demand for medical cannabis as a treatment for psychiatric disorders.

Psychiatric disorders in children and adolescents are particularly prevalent in those with neurodevelopmental disorders, occurring in 40% of children with intellectual disability (ID) [17, 18] compared with 8–20% of children without ID [18, 19]. The total annual cost of ID in Australia is $15 billion [20] and psychopathology directly increases the cost of caring for a child with ID [21]. Psychotropics are commonly prescribed in children for psychiatric disorders and severe behaviour problems associated with neurodevelopmental disorders [22], despite limited efficacy [23], poor adherence [24, 25] and potential adverse effects [26].

The growing community interest in using CBP to treat paediatric neuropsychiatric and neurodevelopmental disorders means physicians are regularly asked about the efficacy and safety of CBP. Although there are several systematic reviews and meta-analyses of CBP in adult psychiatric cohorts [27, 28], there are none in children and adolescents, in whom manifestations of neuropsychiatric disease and response to medication may differ. Thus, data from trials in adults may not be generalisable to younger age groups. In this systematic review we aimed to understand the rationale for use of CBP and evaluate the efficacy of CBP therapies in selected neuropsychiatric and neurodevelopmental disorders in children and adolescents.

## Methods

### Search strategy

A systematic search of MEDLINE, Embase, PsycINFO, and the Cochrane Central Register of Trials was performed in August 2019 and updated in April 2021. This review focused on exogenous cannabinoids and synthetic cannabinoids as treatment for paediatric neuropsychiatric and neurodevelopmental disorders. Searches included terms related to any form of cannabinoid for intended medical use (e.g., marijuana, cannabinoid, cannabidiol, tetrahydrocannabinol, Ajulemic acid, Nabilone, Dronabinol, Nabiximols, Epidiolex^®^ and Sativex^®^). These terms were used in conjunction with any of the following: neuropsychiatric or neurodevelopmental conditions: depression, bipolar disorder, anxiety disorders, psychosis, PTSD, tic disorders/Tourette syndrome (TS), ADHD, ID, Fragile X syndrome (FXS), ASD, and foetal alcohol spectrum disorder (FASD). Publications were limited to papers published since 1980, the year the Diagnostic and Statistical Manual of Mental Disorders (DSM-III) was published, as the DSM-III and subsequent versions have better concordance with present day diagnostic entities than earlier versions. We included publications in ‘humans’ of ‘0–18 years’ or ‘0–17 years’. There were no language restrictions. The search strategy (Online Resource 1) was designed with a research librarian.

**Fig. 1:**
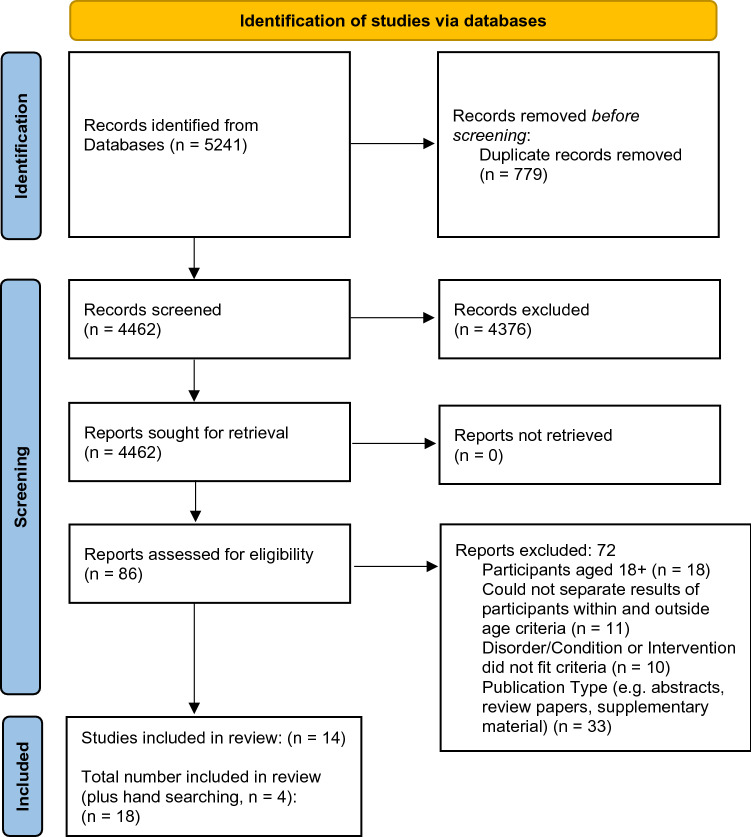
PRISMA 2020 flow diagram. From: Page MJ, McKenzie JE, Bossuyt PM, Boutron I, Hoffmann TC, Mulrow CD, et al. The PRISMA 2020 statement: an updated guideline for reporting systematic reviews. BMJ 2021;372:n71. https://doi.org/10.1136/bmj.n71. For more information, visit: http://www.prisma-statement.org/

Active (incomplete) clinical trials were searched using The WHO International Clinical Trials Registry Platform (trialsearch.who.int/), US National Library of Medicine Clinical Trials (clinicaltrials.gov) and Australian New Zealand Clinical Trials Registry (ANZCTR; anzctr.org.au/) on the 26th of August 2021 using the same terms used in the systematic review. Searches were restricted by age of participants. Only clinical trials that included the neuropsychiatric or neurodevelopmental, conditions specified above were reviewed. As this review was registered before 2021, The Preferred Reporting Items for Systematic Review and Meta-Analysis (PRISMA) 2009 statement [29] guided the current review. However, we have included the PRISMA 2020 flow diagram to include the reasons for exclusion of publications (Fig. 1). The study protocol was registered with PROSPERO on 28/04/2020 (CRD42020153536).

### Inclusion/exclusion criteria

Studies that addressed the efficacy of CBP to treat any of the specified neuropsychiatric or neurodevelopmental conditions in children and adolescents 18 years of age or younger, were included. Epilepsy was excluded because that literature has been summarised in recent reviews [14, 15, 30]. The types of studies eligible for inclusion were meta-analyses, randomised controlled trials (RCT), observational studies, open-label trials, uncontrolled trials, case series, case reports, and *n*-of-1 studies. Efficacy outcomes included changes in psychiatric symptoms, changes in behaviour, or changes in the frequency/severity of behaviour incidents. Safety outcomes included the type and rates of adverse effects. Publications that included only adults or did not provide adequate information to separate the results of participants who were within and outside the age criteria, were excluded.

### Study screening

Titles and abstracts of identified studies were assessed against the eligibility criteria by two independent reviewers, discrepancies were discussed, and a decision was agreed by the two reviewers. For eligible studies, full texts were obtained and assessed and reference lists were screened for other eligible publications. A hand search, including for systematic reviews related to CBP, was conducted and reference lists screened.

### Study quality

One person (LC) extracted the data and another two people (ND, MC) reviewed 50% of the data extraction each. Any discrepancies in data extraction were noted and the reviewers met to discuss. If an agreement could not be made, a third reviewer (LJR) joined the discussion for final decision-making. Minor discrepancies were noted for four of the eighteen papers, resulting in an agreement of 78%. Two reviewers (LJR and EJE) independently assessed the risk of bias and trial quality of each study with 95% agreement, discrepancies were discussed, and a decision was made between the two reviewers. Methodological quality ratings for risk of bias in RCTs were assessed using the Risk of Bias for Randomised Trials (ROBS) 2. Case series and before-and-after intervention studies with no control groups were assessed using the respective National Institute of Health Quality Assessment Tools. Case reports were assessed using the Joanna Briggs Institute Critical Appraisal Checklist for case reports. Case series that included only one or two cases aged 18 years or younger but were written as individual reports were assessed using Joanna Briggs Institute Critical Appraisal Checklist for case reports.

## Results

The initial search strategy identified 4507 articles and 613 duplicates were removed. Of the 3894 articles screened by title and abstract, 56 underwent full-text screening, of which 12 were eligible for inclusion. Review of the references in 23 systematic reviews identified no additional articles. Hand searching identified an additional four articles. The updated search in April 2021 identified 734 articles, from which 166 duplicates were removed, and two additional articles were identified for inclusion.

The most common reasons for excluding articles were that participants were aged 18 years or older; we could not separate the results of participants within and outside age criteria; or CBP use was not intended for medical purposes. A total of eighteen articles was included (Fig. 1).

### Study characteristics

The 18 included articles were published between 1996 and 2021 and included 310 participants aged 3–18 years. One was an RCT, one was an open-label trial, three were observational studies, two were case series, and eleven were case reports. The articles covered eight neuropsychiatric or neurodevelopmental conditions: ASD (*n* = 5), TS (*n* = 3), PTSD (*n* = 3), FXS (*n* = 2), Mood Disorders (*n* = 2), Anxiety Disorder (*n* = 1), FASD (*n* = 1) and ID (*n* = 1). The RCT and open-label trial lasted 8 weeks and 12 weeks, respectively. The duration of the shortest study, a case report, was one week and the longest, also a case report, was 2 years and 5 months. Six studies evaluated the efficacy of cannabidiol (CBD) only, and four evaluated tetrahydrocannabinols (THC) only. The remaining studies evaluated whole cannabis, cannabis extracts or synthesised cannabinoid compounds which typically contained a high ratio of CBD to THC.

Study characteristics, and information on primary outcomes, CBP, efficacy and safety of the eighteen articles are shown in Table 1. Online Resource 2 contains: (i) detailed information for each of the 18 articles (including: participants secondary conditions, CBP administration and dosage, outcome measures, results, completion rates, reasons for termination, concomitant medication and adverse events experienced by participants), (ii) risk of bias and trial quality and (iii) clinical trials in progress. Online Resource 3 provides a summary and quality of evidence of case series and case reports. Online Resource 4 provides a list of the publications that were excluded after full-text review because the results of participants within and outside age criteria could not be separated.

**Table 1:** Summary of the characteristics, efficacy, and safety for studies of cannabinoid-based products (CBP) in the treatment of neurodevelopmental and neuropsychiatric disorders in children and adolescents

Author		Aim	Country^a^	Study design	Study duration	Participants N; (% male); Age	CBP	Clinical summary	Adverse events	Safety summary
Anxiety disorders										
Klier et al.		Present a case with comorbid anxiety disorders (and Crohn’s disease) who was treated with CBD for 26 weeks	Austria	Case report	26 weeks	N = 1 (0%); 14	CBD oral capsules	Suggests CBD might improve severity of symptoms and some behaviours, but not remission	Unknown	Safety not mentioned
(Also see Laczkovics et al. under “Mood disorders”)										
ASD										
	Aran et al.	Evaluate the safety and efficacy of cannabidiol-rich cannabis in children with ASD and severe behavioural problems	Israel	Retrospective before and after study with no control group	10 months	*N* = 60 (83%); 5–17	Whole plant extracts dissolved in olive oil. CBD and THC (initial ratio of 20:1). Variability in the strain and dose across participants	Suggests that CBD and THC (20:1) might improve core ASD symptoms in some children with ASD (communication and anxiety) and severe behaviour problems. Positive responses were slightly higher when examined only in participants with idiopathic ASD	Yes	Unclear how many participants experienced more than one adverse event. Strains with high dose of THC might cause severe psychotic episode
	Bar-Lev Schleider et al.	Characterize the patient population receiving medical cannabis treatment for autism and evaluate the safety and efficacy of this therapy	Israel	Prospective before and after study with no control group	6 months	*N* = 188 (82%); ≤ 18	CBD and THC (20:1). 94.7% of the patients: cannabis oil (extract of a high CBD strain dissolve in olive oil; 30% CBD & 1.5% THC) applied under the tongue; 7 (3.7%) oil and inflorescence; 3 (1.5%) only inflorescence	This study suggests cannabis treatment may improve a range of symptoms (e.g., seizures, tics, depression, restlessness, and rage attacks), and quality of life for individuals with ASD	Yes	Cannabis appears to be well-tolerated and safe treatment for ASD. The adverse effects reported by the patients and parents were moderate and relatively easy to cope with. High level of compliance (< 15% stopped treatment at 6 months follow-up; and < 5% stopped treatment due to adverse events). Suggests a careful titration schedule is important to maintain a low adverse effects rate and increase the success rate
	Fleury-Teixeira et al.	Analyse the effects of the compassionate use of CE given to ASD patients	Brazil	Prospective before and after study with no control group	6 months (*n* = 1); 9 months (*n* = 14)	*N* = 18 (72%); 6–17	CBD-enriched Cannabis sativa extract (CE). CBD and THC (75:1). Oral capsules containing 25 or 50 mg of CBD and ∼ 0.34 or 0.68 mg of THC	Suggests CBD and THC (75:1) might improve multiple ASD symptoms of children with ASD (including epileptic and non-epileptic patients)—particularly ADHD, Sleep Disorders and Seizures; and without the typical side effects found in medicated ASD patients. Also suggests improvements continue after reducing/discontinuing other medications	Yes	Suggests non-epileptic patients had fewer adverse effects compared to currently available therapies. Not all benefited equally from the treatment, with those receiving concomitant medication (including at least one antipsychotic) reported negative results (*n* = 4/18)—suggesting possible undesirable drug interactions and potential risk when CE is used in a drug combination. Suggests gradual increase in the dosage of CE in patients receiving many drugs, and cautiously evaluate the possibility of partial/complete withdrawal of previously prescribed drugs
	Kurz and Blaas	Investigate whether dronabinol (∆-9-THC) could safety be used in autism and what outcomes can be achieved within 6 months	Austria	Case report	6 months	*N* = 1 (100%); 6	THC Dronabinol drops (dronabinol solved in sesame oil)	Suggests THC improves hyperactivity, lethargy, stereotype, irritability, and inappropriate speech of a child with ASD. THC may be an effective additional support (with CBT) and be better tolerated than many antipsychotic drugs	No	Larger controlled studies needed to explore the safety of THC
	Ponton et al.	Describe the novel use of a CBD-based extract on a patient with ASD	Canada	Case report	2 years and 5 months	*N* = 1 (100%); 15	Cannabinoid extracts. CBE (60 mL bottle of 1:20 CBE (0.001% THC, 0.02% CBD), with olive oil carrier)	Suggests CBE improves social communicative and behavioural ASD symptoms, anxiety, sleep dysregulation and weight; and at a lower dose than previously studied. These improvements could lead to improved daily functioning and better QoL	No	No side effects reported Rigorous, controlled clinical trials are needed to further establish safety, especially long-term safety, optimal dosing, and efficacy, including further delineation of the effect of CBE on core versus associated ASD symptoms
FASD										
	Koren et al.	Analyse the effect of cannabis in children and young adults diagnosed with FASD	Israel	Case series	2 years; N/A	*N* = 2 (100%); 5yrs + 4 months, 12	CBD and THC. CBD oil (5yo: 20% CBD, 0.2% THC; 12yo: 15% CBD, 1% of THC)	Suggests CBD oil may improve disruptive symptoms such as aggression and impulsivity, of children with FASD	No	A well-controlled study is needed to explore the safety of CBD CBD as a pharmacological solution for disruptive behaviour may be advantageous, as second-generation antipsychotic drugs have risk of adverse effects (CNS effects, weight gain, complex metabolic changes)
FXS										
	Heussler et al.	Evaluate the safety, tolerability, and initial efficacy of a transdermal CBD gel (ZYN002) in a paediatric population with FXS	Australia	Open-label multi-site trial	12 weeks	*N* = 20 (75%); 6–17	CBD Transdermal Gel (ZYN002)	Suggests CBD Transdermal Gel may result in statistically significant reduction in anxiety, emotional and behavioural symptoms for children and adolescents with FXS	Yes	Majority (70%) of adverse events were mild and resolved by the end of the 12-week treatment period Results suggest ZYN002 CBD gel is clinically safe (and tolerated) for the treatment of FXS patients
	Tartaglia et al.	Present the cases of one child and two adults with FXS who were treated with various oral botanical CBD + solutions	USA	Case report (1 eligible participant in case series of *n* = 3)	~ 1 year 3 months	*N* = 1 (100%); 3 years + 6 months	CBD and THC. Oral paste (18–23.5% CBD and trace amounts (0.03%) of THC)	Suggests CBD may provide therapeutic effects for children with FXS (e.g. language, social and motor skills, anxiety, sleep, adaptive function)	No	Suggests CBD is generally well tolerated among individuals with FXS. Open-label trials and placebo-controlled trials should be conducted
ID										
	Efron et al.	Investigate the feasibility of conducting a RCT of CBD to reduce severe behavioural problems in children with ID	Australia	RCT	8 weeks	*N* = 8 (*a*: 63%; *b*: 58.3%); 8–16	CBD oral solution: 98% CBD 100 mg/ml in grapeseed oil	Suggests CBD may significantly improve behaviour in children with ID compared to a placebo	Yes	Suggests CBD is feasible and acceptable for patients with ID and severe behavioural problems and their families. Proposes future research develop a specific measure that succinctly focuses on common adverse events seen with CBD (MOSES was suboptimal for this study)
Mood disorders										
	Gruber et al.	Present 5 cases in which the evidence seems clear that marijuana produced a direct antidepressant effect	USA	Case report (1 eligible participant in case series of n = 5)	Started in 8th grade	*N* = 1 (100%); 16	marijuana	Suggests marijuana may provide relief of depressive symptoms and may be more effective than traditional antidepressants (e.g., fluoxetine)	Unknown	Adverse effects not mentioned
	Laczkovics et al.	Present a case with severe depression and other conditions/disorders who was administered CBD capsules for over 6 months	Austria	Case report	> 6 months	*N* = 1 (100%); 16.9	CBD oral capsules	Suggests CBD capsules improve depressive and anxiety symptoms (especially social anxiety and withdrawal), even after the cessation of antidepressant medication	No	Safe, well tolerated and good adherence
PTSD										
	Bolsoni et al.	Assess the effects of a 7-day oral treatment with CBD on the reconsolidation of memories related to the traumatic event (acute sexual violence)	Brazil	Case report	7 days	*N* = 1 (0%); 15	CBD oral capsules (CBD powder: 99.9% purity dissolved in corn oil)	Suggests CBD may not prevent an increase in anxiety after recollection of a traumatic event or prevent the development of PTSD; but it may interfere with the reconsolidation of traumatic memories	Unknown	Adverse effects not mentioned
	Lorenz et al.	Discuss the application of ∆-9-THC in 8 child case studies suffering from various conditions	Germany	Case report (× 2)	N/A; 3 months	*N* = 2 (50%); 3 years + 10 months, 11	THC oral	Suggests THC may improve posttraumatic reactions (and possibly eating disorders) among children. Optimal THC dose likely to depend on patient’s indication	No	Adverse effects of THC in children differs to those in adults
	Shannon et al.	Provide evidence that CBD is effective as a safe alternative treatment to traditional psychiatric medications for reducing anxiety and insomnia secondary to PTSD	USA	Case report	5 months	*N* = 1 (0%); 10	CBD oil (capsule) and CBD liquid (sublingual spray)	Suggests CBD oil can reduce anxiety and insomnia as part of PTSD among children	No	Suggests CBD oil is generally safe with minimal side effects Long-term effects are yet to be studied
TS										
	Hasan et al.	Examine the clinical effects of ∆-9-THC as an add-on treatment in an adolescent experiencing severe TS plus ADHD treated with stimulants	Germany	Case report	9 weeks	*N* = 1 (100%); 15	THC (∆-9 THC)	Suggests THC might be an effective treatment or add-on therapy for adolescents with severe TS, who do not respond to neuroleptics alone	No	Suggests THC might improve TS with limited adverse effects; and might be an effective add-on therapy to prevent tic-exacerbations in those taking neuroleptics and stimulants
	Jakubovski and Muller-Vahl	Examine the improvement in speech fluency, social impairment, and patients’ quality of life different cannabis-based medicines have on two cases of TS patients (suffered from disabling vocal blocking tics and palilalia)	Germany	Case report (1 eligible participant in a case series of *n* = 2)	1 year (and 8 months after 1 year)	*N* = 1 (100%); 16	CBC and THC vaporized dronabinol	Suggests CBD (medical cannabis or dronabinol) may improve simple and complex motor and vocal tics; overall symptomology associated with TS, including comorbid conditions, and improve QoL of individuals with treatment-resistant TS	Yes	No severe side effects reported
	Muller-Vahl et al.	Cannabinoids: possible role in pathophysiology and therapy of Gilles de la Tourette syndrome	Germany	Case series	N/A; > 3 years	*N* = 2 (100%); 16, 18	marijuana cigarettes	Suggests marijuana use may reduce both motor and vocal tics among individuals with TS	No	No severe side effects reported

*CBD* cannabidiol, *CE* Cannabis sativa extract, *PEA* palmitoylethanolamide, *QoL* Quality of Life

^a^When country of recruitment was not specifically mentioned within the paper, the location was taken from other information within the paper (e.g., authors affiliation, ethics committee)

Below, a brief description of each condition is followed by the evidence from the eighteen included studies and the fourteen relevant forthcoming clinical trials. The findings from the review are presented by condition and include information on the rationale for use of CBP, the evidence by type of CBP, the quality of evidence/risk of bias and a description of active (incomplete) trials.

### Autism spectrum disorder

#### Rationale

The ECS is thought to play a role in social interaction, emotional responses, and behavioural reactivity in ASD [31]. Findings from ASD mouse models suggest that increasing AEA activity at CB_1_ improves function in ASD, particularly social interaction [32, 33]. In human studies, children with ASD had lower plasma and serum levels of AEA compared to healthy controls [34, 35]. Together these studies suggest impaired AEA signalling may be involved in the pathophysiology of ASD [36]. CBD, one of the primary cannabinoids in the cannabis plant, inhibits the reuptake and degradation of AEA and hence has the potential to elevate AEA levels [36]. Additionally, a clinical study showed that CB_2_ is upregulated in peripheral blood mononuclear cells of children with ASD compared to healthy controls and the authors proposed that CB_2_ is a potential target for ASD treatment [37]. Consequently, the mechanism by which cannabinoids could be used to treat ASD may be through the synthetic modulation of the ECS [38].

A prospective observational study of children and young adults with ASD (*n* = 53; aged 4–22) suggested CBD could improve hyperactivity, sleep, self-injury, and anxiety. Although efficacy did not differ statistically from conventional treatments (e.g., methylphenidate, melatonin, aripiprazole, selective serotonin reuptake inhibitors), non-inferiority of CBD was observed [39]. A three-arm RCT, compared the efficacy of whole plant cannabis extract (20:1 CBD/THC ratio), pure CBD and pure THC (20:1 ratio) and an oral placebo among children and young adults with ASD (*n* = 150; aged 5–21) [40]. No severe or serious adverse events were reported, with somnolence being the most reported mild adverse event. This trial suggested that CBD may improve disruptive behaviours in ASD [40]. These two trials were excluded from the current review as they included participants out of the age range.

#### Evidence in children and adolescents

We identified five published studies evaluating the efficacy of CBP in children and/or adolescents with ASD. These include three before-after observational studies with no control group [41–43] and two case reports [44, 45] (Table 1). The symptoms that improved most often across the studies were maladaptive behaviours (e.g., aggression, irritability, and hyperactivity), and core impairments of ASD (social skills, social communication, and repetitive behaviours). Some improvements were noted in sleep, lethargy, anxiety, mood, quality of life, adaptive behaviours, and cognition (Table 1). Adverse effects were mostly mild to moderate and transient. Concomitant medications were reported in four of the five studies, in about two-thirds of the cohort [41–45] (Online Resource 2).

In the three before-and-after observational studies [41–43] and a case report [45] whole plant extracts with high doses of CBD and THC or CBD and THC isolates were used (Table 1). The three observational studies included administration of whole plant extract (*n* = 60) [41], cannabis oil (*n* = 188) [42] and cannabis Sativa extract (CE) (*n* = 18) [43], CBD/THC ratios were: 20:1; 20:1; and 75:1, respectively. Primarily based on standardised caregiver reports, each treatment improved ASD symptoms. Specifically, in the retrospective study in which whole plant extracts were administered for an average of 10 months improvements were reported in behaviour (61%), anxiety (39%) and communication (47%) and these rates were slightly higher when examined in participants with ASD not associated with another diagnoses [41]. In the study in which sublingual cannabis oil (30% CBD, 1.5% THC) was used, more than 80% of parents reported moderate/significant improvement in their child’s global assessment, and significant improvements in quality of life, mood, sleep, and the ability to perform daily activities (e.g. dress and shower independently) [42]. The third study, in which oral capsules of CBD-enriched CE were administered (CBD dose: 3.75–6.45 mg/kg/day; THC dose: 0.05–0.09 mg/kg/day) improvements were reported in more than one of the eight ASD symptoms/signs evaluated: 60–80% of participants had some level of improvement in ADHD, motor deficits, communication and social interaction, cognitive deficits and/or sleep disorders [43] (Table 1 and Online Resource 2). Adverse events reported in these observational studies were mild/transient, the most common being sleep disturbance, restlessness, change in appetite, and nervousness [41–43] (Online Resource 2). Despite improvements in ASD symptoms, one case receiving the CE treatment developed worsening psycho-behavioural aspects. This study only reported side effects in children receiving concomitant medication and the authors highlighted the possibility of negative CE-drug interactions, especially among participants taking antipsychotic medications [43] (Table 1 and Online Resource 2). Dropout rates for these observational studies were between 12% and 27%, predominately due to low perceived efficacy and/or adverse events (e.g., insomnia, irritability, worsening psycho-behavioural crisis, and/or increased heart rate [43]) (Online Resource 2). The case report is described in Online Resource 3.

See Online Resource 3 for a summary of the case report that trialled Delta-9-tetrahydrocannabinol (THC) [44].

#### Quality of evidence/risk of bias

The five studies were uncontrolled; the assessors were typically parents and treating physicians and were not blinded to the intervention, which increased the risk of bias. Previous studies suggest 20–50% of parents report a positive response when their child with ASD receives a placebo [46], so findings from uncontrolled studies must be interpreted with caution. There were considerably more males than females in each study, which fits with the gender ratio in ASD but makes the findings difficult to generalise to females. Outcomes and outcome measures varied widely across the studies and standardised assessment tools were not always used, making it difficult to compare findings between studies or conduct meta-analyses. There was also variability in the cannabinoid strain used, e.g., THC, CBD. A potential conflict of interest was reported in one study [42] and was unclear in another [44].

Although these preliminary studies suggest CBP might improve some ASD symptoms, the high risk of bias in the five studies (Online Resource 2) indicate the need for RCTs.

#### Active clinical trials

Seven clinical trials (RCT (*n* = 4), open-label (*n* = 3)) are underway in Australia (*n* = 2) and the USA (*n* = 5) to assess the efficacy of CBP among children and/or adolescents with ASD (Online Resource 2).

### Intellectual disability

#### Rationale

About 40% of children with ID experience severe emotional and behavioural problems, including irritability, aggression, and self-injurious behaviour. Anxiety is a driver of severe behavioural problem in individuals with ID and CBD has anxiolytic effects [47, 48]. However, it is not known whether ID is associated with alterations in the ECS. There is growing interest from parents of children with ID to determine whether CBP can reduce behaviour problems in children and adolescents with ID [49, 50].

#### Evidence in children and adolescents

An 8-week, double-blind, placebo-controlled, parallel-group, RCT was piloted to investigate the feasibility of the protocol for evaluating CBD in children and adolescents with ID and severe behavioural problems (*n* = 8, aged 8–16 years old) [50] (Table 1). The treatment group received CBD oil (maintenance dose of 20 mg/kg/day, with a ceiling dose of 1000 mg/day). Outcome measures included the Aberrant Behaviour Checklist—Irritability subscale (ABC-I, primary outcome); Child & Adolescent Scale of Participation; Child Health Utility 9D; Sleep Disturbance Scale for Children; Adult Quality of Life Total; Beach Center Family Quality of Life Depression Anxiety Stress Scale; and Autism Parenting Stress Index and were completed in the treatment (*n* = 3) and placebo (*n* = 4) groups (Online Resource 2). An efficacy signal was found on the ABC-I in favour of CBP treatment. Using the Monitoring of Side Effects Scale (MOSES), some minor adverse effects were experienced by participants in both groups (e.g. abdominal pain, constipation, tinnitus, restlessness, drowsiness), but no severe adverse events were reported [50] (Online Resource 2). For future trials the authors suggested use of a measure that focuses on common adverse events seen with cannabis/CBD for medical use instead of the MOSES; a decrease in the number of outcome assessments, laboratory tests and study visits; and flexibility for parents to complete some of the questionnaires at home.

#### Quality of evidence/risk of bias

There was a low risk of bias in the pilot RCT design. However, the sample size was too small to evaluate the significance of the positive signal for CBD. Also, all participants randomised into the CBD group had ASD compared to only one of four in the placebo group and the primary outcome measure was designed to assess behaviours in people with ASD (Online Resource 2).

#### Active clinical trials

A multi-site, double-blind, parallel-group, RCT to extend the pilot [50] is underway in Australia to evaluate the efficacy of CBD isolate in oral solution in reducing severe behavioural problems among children/adolescents with ID (Online Resource 2).

### Fragile X syndrome

#### Rationale

FXS is caused by a mutation of the fragile X Messenger Ribonucleoprotein 1 gene (FMR1), resulting in absent or reduced production of the protein FMRP which is required for brain development. FMR1 knock-out mice have the same symptoms as people with FXS. Studies with these mice suggest that reduced expression of FMR1 dysregulates the ECS and disrupts the excitatory and inhibitory neurotransmission balance via the post synaptic release of endogenous cannabinoids, AEA and 2-AG, that stimulate CB_1_ receptors that occur throughout the CNS [51–53]. It is thought that CBD may help restore the excitatory and inhibitory balance by increasing AEA and 2-AG availability [52–55]. Two case reports in adults with FXS demonstrated reductions in anxiety and social avoidance and improvements in sleep, feeding, motor coordination and language skills following oral administration of CBD-enriched (CBD+) solutions [56]. However, these case reports relied on parental reports, did not include control groups and, include inconsistencies in the quality, purity, and administration of CBD, making it challenging to reach conclusions [56].

#### Evidence in children and adolescents

One uncontrolled, open-label, before-and-after trial [57] and a case report [56] suggest that CBD may benefit children and adolescents with FXS (Table 1). The 12-week open-label multi-site trial, which administered transdermal CBD gel (50 mg/day (*n* = 1); 100 mg/day (*n* = 3); 250 mg/day (*n* = 16)) to 20 children (aged 6–17 years), reported a significant reduction in anxiety and behaviour symptoms. The Anxiety, Depression and Mood Scale (primary outcome) showed statistically significant reductions in the mean total score and several subscales (manic/hyperactive behaviour, social avoidance, general anxiety, and compulsive behaviour) from screening to week 12. No improvement was found on the depressed mood subscale. Several secondary measures also reported significant reductions in mean scores—The Aberrant Behavior Checklist—Community for FXS: Clinical Global Impression Scale—Severity and Improvement; The Pediatric Quality of Life Inventory; The Pediatric Anxiety Rating Scale; and three Visual Analogue Scales, hyperactivity/impulsivity, tantrum/mood liability, and anxiety (Online Resource 2). Two of the twenty participants withdrew from the study on day 63 (dose: 100 mg/day) and day 64 (dose: 50 mg/day). These participants were siblings: one withdrew due to adverse effects, worsening pre-existing eczema, and the other withdrew for administrative reasons. Seventeen of the twenty participants (85%) reported at least one adverse event, the most common being: gastroenteritis (*n* = 5), disorders at the application site (*n* = 2), vomiting (*n* = 2) and upper respiratory tract infection (*n* = 2). No serious adverse events were reported [57] (Online Resource 2). See Online Resource 3 for a summary of the case report.

#### Quality of evidence/risk of bias

The two studies that examined the efficacy of CBP in FXS both used CBD; however, the open-label trial used a transdermal gel, and the case report used an oral paste. Both studies were uncontrolled, and assessors were not blind to the intervention, which increased the risk of bias (Online Resource 2). There were no other quality concerns with the before-and-after trial. See Online Resource 3 for a summary and quality of evidence of the case report.

#### Active clinical trials

Two active clinical trials are evaluating the efficacy of ZYN002, a transdermal CBD gel, for treatment of FXS. The first is an extension of the uncontrolled open-label before-and-after trial discussed above [57], intended to evaluate the long-term safety and tolerability of ZYN002 in children with FXS. The second trial is a phase 3 multicentre RCT using the same CBD product and similar dose that will be conducted by the same authors and funded by Zynerba Pharmaceuticals (Online Resource 2).

### Mood disorders

#### Rationale

Depression, mania, and bipolar spectrum disorders are conditions for which novel treatments are in demand, especially when there are recurrent or refractory episodes of illness. The role of the ECS has been studied using a knock-out mouse model. Absence of the CB_1_ gene results in deficiency of CB_1_ receptor signalling and aggressive behaviour and an anhedonic state in the mice, both of which are core depressive symptoms in human adolescents [58]. In humans, antagonism at CB_1_ receptors with rimonabant (an inverse agonist of CB_1_) is associated with depressive symptoms in adult humans [59]. The antidepressant effect of CBD may involve a CB_1_-mediated increase in serotonin levels involving the 5HT1A receptor [60, 61].

Clinical practice guidelines for the assessment and treatment of mood disorders note that the evidence for efficacy of CBD in depression remains unproven [62]. Cross-sectional studies in Australia [63] and the USA [64] suggest a sizeable proportion of adults self-medicate with CBD to treat their depression, despite unproven efficacy [65, 66]. CBD was deemed ineffective in two manic patients, one of whom received adjunctive olanzapine [67]. Two small-scale controlled trials (*n* = 8; *n* = 13) conducted in the 1970s did not find significant effects of THC on depressive symptoms among individuals with unipolar and bipolar depressive disorders [68]. One double-blind placebo-controlled cross-over trial of THC in 34 patients with cancer reported improvements in depression. However, this study had methodological limitations and confounding effects of cancer.

#### Evidence in children and adolescents

There is no RCT, one case series [69], and one case report [70] evaluating the efficacy of CBP for mood disorders in children (Table 1). See Online Resource 3 for a summary of these reports.

#### Quality of evidence/risk of bias

The case series and case report offer no evidence for the efficacy of CBP in improving mood disorder symptoms (Online Resource 2). See Online Resource 3 for a review of the quality of evidence of these reports.

#### Active clinical trials

A phase 2, double-blind, randomised placebo-controlled trial was registered in Australia in 2016 to examine the safety and efficacy of CBD capsules in managing mood disorders in males aged 6–26 years old who use cannabis (Online Resource 2).

### Anxiety disorders

#### Rationale

About half of cannabis users in the general population report using cannabis for anxiety [48]. Systematic reviews of animal and human studies evaluating CBD (and THC) as potential treatments for anxiety disorders [28, 47, 48, 71–73] suggest that CBD is anxiolytic, and THC is anxiogenic [47, 48, 71–73]. Although it is thought that lower doses of THC are anxiolytic, higher doses are anxiogenic [48]. The dose–response for CBD appears to follow a bell-shaped curve, with less effective results at lower and higher doses [47, 72]. CB_1_ receptors, 5-HT1A receptors and TRPV1 receptors are likely mediators of CBD’s anxiolytic effects [47, 72, 73]. The ineffectiveness at higher dosage may be due to activation of TRPV1 receptors, which have anxiogenic effects [47]. The anxiogenic-like effects of THC diminish when CBD is co-administered with THC, suggesting that CBD alleviates some of THC’s anxiogenic-like effects [48, 72, 73].

There is growing literature on the complex circuitry underlying the modulation of anxiety and anxiety disorders by the ECS (for a detailed description see [74]). An example of one circuit includes the basolateral amygdala (BLA) to prefrontal cortex (PFC) pathway. The BLA plays an important role in inducing anxiety in response to environmental cues. The anxiolytic effects of THC are associated with reduced connectivity between the BLA and PFC [74, 75]. It is thought that a persistent weakening of the ECS tone could increase the BLA-PFC pathway connectivity and progress a stress response into an anxiety disorder [74, 76].

Some studies suggest CBD may improve symptoms of anxiety compared to placebo. However, most of these studies had small sample sizes, a single-dose design, or short duration, and some included people without confirmed anxiety disorders [28, 71, 77]. There has been limited research into the efficacy of nabilone (a synthetic THC) and findings are mixed [28]. Animal studies show variable results, likely due to use of different methodologies (e.g., variations in the animal studied; CBD strain, dosage, route, and pattern of administration, etc.) [48, 72, 73]. Although CBD may be effective for anxiety disorders, more RCTs are needed.

#### Evidence in children and adolescents

A single case report has been published on CBD administration for a child with an anxiety disorder [78] (Table 1). See Online Resource 3 for a summary of this case report.

#### Quality of evidence/risk of bias

This single case report is insufficient to determine the potential efficacy of CBP in the treatment of anxiety in children or adolescents. See Online Resource 2 for the critical appraisal checklist, and Online Resource 3 for a summary and quality of evidence of the case report.

#### Active clinical trials

A 12-week open-label pilot study of the safety, tolerability, and efficacy of CBD for anxiety disorders in people aged 12–25 years was registered in Australia in 2018 but is not yet actively recruiting (Online Resource 2).

### Post-traumatic stress disorder

#### Rationale

Neuroimaging studies show the amygdala [79] and hippocampi [80] are hyper-responsive and the medial prefrontal cortex is hypo-responsive during symptomatic PTSD and the findings are associated with symptom severity [79, 81]. Although hyperactivity of the amygdala is found in other anxiety disorders, hypo-activation of the prefrontal cortex is not, suggesting this may be specific to PTSD [81]. The concentration of endogenous cannabinoids, anandamide and 2-arachidonoyl glycerol, is reduced in people with PTSD [82, 83]. Anandamide decreases the activity of inhibitory interneurons in the amygdala, which increases the activity of output neurons, which play a role in memory extinction [84]. In healthy people, THC increases prefrontal cortex and hippocampal activation during extinction memory recall and attenuates amygdala activity during early extinction learning [85]. Together, these studies suggest the ECS may be a promising target for intervention in PTSD.

A systematic review identified ten studies published to 2018 that examined the effectiveness of CBP in treating PTSD in mostly adult cohorts. These studies suggest CBP may help reduce global PTSD symptoms, sleep disturbances and nightmares [86]. Only one study was an RCT, and this cross-over trial evaluated the efficacy of nabilone in reducing PTSD-related nightmares in ten male military personnel. A statistically significant reduction in the severity of PTSD-related nightmares was documented after 10-weeks of nabilone, as were improvements in general wellbeing [87]. This RCT and other studies identified in the systematic review were of low quality with medium to high risk of bias, making it difficult to draw definitive conclusions [86].

#### Evidence in children and adolescents

Three publications, all case reports, focused on the effects of CBD/THC on a total of four children/adolescents with PTSD [88– 90] (Table 1). For a summary see Online Resource 3.

#### Quality of evidence/risk of bias

The quality was poor for the two cases that were administered THC [89]. The two case reports that described patients who were administered CBD were of a higher quality [88, 90] (Online Resource 2). See Online Resource 3 for more details.

#### Active clinical trials

Although three RCT are registered for adults, no active clinical trials are underway regarding CBD in children/adolescents with PTSD.

### Tourette syndrome

#### Rationale

The underlying cause of TS remains unknown. However, research suggests endocannabinoid receptors have a role in motor function and may be involved in TS pathology and that cannabinoids might reduce the frequency and intensity of tics and premonitory urges [91–94]. Furthermore, a recent study found adults with TS have higher cerebrospinal fluid levels of endogenous cannabinoids, AEA and 2-AG, than typically developing controls. It is not clear whether the increased levels of endogenous cannabinoids have a primary role in the aetiology of TS or whether they are a secondary response to dopaminergic abnormalities [95].

Observational and retrospective studies suggest CBP may reduce tics, urges, compulsive behaviours, and ADHD symptoms in people with TS [92, 96, 97]. Some case reports in patients with treatment-resistant TS suggest that an oro-mucosal spray that contains THC and CBD (e.g., Nabiximols, Sativex), may improve tic severity and quality of life [97, 98]. Recent reviews [91, 99–101] identified only two RCTs of CBP for treatment of adults with TS; a double-blind placebo-controlled cross-over single-dose trial [102], and a randomised, double-blind placebo-controlled 6-week trial [93]. Both RCTs had small sample sizes, short durations, a high or unclear risk of bias [100] and some systematic reviews stated that the effectiveness was unclear [101]. Overall, there is limited evidence that CBDs benefits some, but not all, individuals with TS. More research is required to determine the efficacy of CBD for reducing tic severity and urges among individuals with TS [99].

#### Evidence in children and adolescents

Two case reports [103, 104] and one small case series [105] have been published on use of CBP, THC (*n* = 1) [103], vaporised dronabinol (22% THC and 1% CBD; *n* = 1) [104], and marijuana cigarettes (*n* = 2) [105], respectively for adolescents with TS (Table 1). See Online Resource 3 for a summary of these reports.

#### Quality of evidence

None of these studies included controls, making them at high risk of bias (Online Resource 2). Other confounders include comorbidities, use of other medications, self-report, and inconsistent outcome measures (Online Resource 2). See Online Resource 3 for a summary of the quality of the reports.

#### Active clinical trials

No active clinical trials are registered regarding CBP for intended medical use, among children/adolescents with TS.

### Foetal alcohol spectrum disorder

#### Rationale

FASD is a severe neurodevelopmental disorder which results from brain injury caused by prenatal alcohol exposure (PAE) [106, 107]. Various mechanisms, including endocannabinoid mechanisms, likely contribute to the neurological damage associated with FASD [108]. PAE interacts with the glutamate or gamma-aminobutyric acid (GABA) transmitter systems, leading to suppressed neuronal activity, and may trigger widespread apoptotic degeneration of neurons [108, 109].

Despite limited data on effectiveness, stimulants (e.g., methylphenidate, amphetamine) for ADHD symptoms and atypical antipsychotics (e.g., risperidone) for aggressive and defiant behaviours are the commonly used pharmacological therapies among individuals with FASD [110]. A preclinical study in mice suggests that CBD may counteract cognitive impairments caused by prenatal and lactation alcohol exposure [111]. To our knowledge, there has been only one study in the use of CBP for FASD, a case series of two children and three young adults with disruptive behaviours [112]. Parents reported reductions in disruptive symptoms such as restlessness, aggression, and impulsivity after treatment with CBD and no serious adverse effects. The heterogeneous presentation of FASD, high rate of comorbidities, and overlapping symptoms between FASD, ASD, ADHD and ID will make clinical studies attempting to determine the effectiveness of CBD treatment in individuals with FASD a challenge [113].

#### Evidence in children and adolescents

One case series, with two children (a 5-year 4-month-old male; and a 12-year-old male), has been published on the use of CBD oil (20% CBD, 0.2% THC: and 15% CBD, 1% THC, respectively) in FASD [112] (Table 1). See Online Resource 3 for a summary of the case series.

#### Quality of evidence/risk of bias

The case series did not adequately describe the patients’ history, current clinical condition or intervention provided, supporting the need for an RCT (Online Resource 2).

#### Active clinical trials

No active clinical trials are underway regarding CBP for intended medical use, among children/adolescents with FASD, however children with FASD, ID and severe behavioural problems are eligible for inclusion in an Australian trial [48].

### Attention deficit/hyperactivity disorder

Although eight of the eighteen included articles had at least one child/adolescent with comorbid ADHD [42, 43, 50, 57, 69, 103, 105, 112] only one of these trials examined the role of CBP for management of ADHD symptoms [43] (see Table 1, and Online Resource 2). We did not find any published or registered trials of CBP exclusively for children/adolescents with ADHD. Based on recent reviews [27, 113–115] there is only one RCT of a CBP in adults with ADHD [116]. In this pilot study, 30 adults with ADHD were randomly assigned Sativex oromucosal spray (THC and CBD in approximately 1:1 ratio) or placebo for 6 weeks. Cognitive performance, activity level (head movements) and ADHD symptoms (inattention, hyperactivity/impulsivity, and emotional lability) were assessed. Improvement was reported only for hyperactivity/impulsivity, but this was not statistically significant after controlling for multiple comparisons [116].

#### Active clinical trials

We found no clinical trials registered that examine the efficacy of CBP in paediatric ADHD.

### Psychosis/schizophrenia

A meta-analysis of 18 studies reported an increase in AEA in the CSF and blood of people with schizophrenia compared to typically developing controls. The review also found higher ECS tone in early stage psychosis that was inversely associated with symptom severity and normalised with treatment [117]. There are no published trials of medicinal use of cannabinoids for children/adolescents with psychosis or schizophrenia. The lack of trials is not surprising given cannabis use in adolescence can increase the risk of developing psychosis and earlier onset of psychotic symptoms. A meta-analysis found that the increased risk of psychosis from adolescent cannabis use is moderated by the age at onset of cannabis use, frequent cannabis use, exposure to early life trauma, concurrent use of other substances and genetic factors [118]. A recent review identified six RCTs of CBP for symptoms of schizophrenia or psychosis in adults. Meta-analysis showed mixed results for the effectiveness of CBD as an adjunct treatment for positive symptoms and no evidence for negative symptoms [27].

A small trial of intravenous THC is underway to evaluate the efficacy of CBD for adults with psychosis [65], adults with early psychosis and comorbid cannabis use [119], and young people (aged 18–35 years) at high risk for psychosis [115]. There are no trials of medicinal use of CBP for children/adolescents with psychosis.

#### Active clinical trials

There are two registered RCTs involving children/adolescents to assess the effect of CBD on psychotic symptoms. These studies include administration of oral CBD capsules to people aged 12–25 years with psychotic symptoms who are identified as having ultra-high risk of psychosis (Australia, not yet recruiting); and administration of CBD in oral solution to 16–30-year-olds with early psychosis (USA, recruiting) (Online Resource 2).

## Discussion

There is growing interest in understanding the efficacy of CBP to treat neuropsychiatric and neurodevelopmental disorders in children and adolescents. Although there is a biological rationale for trialling CBP in a range of disorders, this systematic review found a paucity of high-quality clinical trials in children/adolescents, including only one RCT and one open-label trial. Given this is a developing area of research, we included uncontrolled trials, case series and case reports in our review to provide a thorough summary of the existing knowledge and identify ways forward.

### Efficacy and risk of bias summary

Our systematic review identified eighteen articles addressing eight neuropsychiatric or neurodevelopmental conditions with 310 participants. The condition most studied was ASD. However, there were no RCT’s conducted only with children (see Online Resource 4 for RCT conducted with children and adults), only before-and-after observational studies, non-controlled case series, or case reports [41–45]. Consistent with research conducted with adults with ASD, the existing CBP trials in children suggest these products might help improve disruptive and core ASD behaviours [120]. However, a wide range of target behaviours and outcome measures were used and studies were small and uncontrolled. RCTs are needed to determine if CBP are an effective treatment for ASD. The only RCT identified in this review examined the feasibility of a study protocol for evaluating the efficacy of CBD for reducing severe behaviour problems in children and adolescents with ID [50]. The study was deemed feasible and there was a positive signal for treatment effect, however, there were only eight participants and the authors have commenced a larger RCT.

We identified one open-label trial in FXS [57] that found CBD reduced a range of emotional and behavioural problems [56, 57]. These findings are consistent with case reports in adults with FXS [56]. However, without a control group these findings must be interpreted with caution. There was less evidence for the efficacy of CBP in improving symptoms of paediatric PTSD (four case reports), anxiety disorders (one case report), mood disorders (two case report), TS (two case reports and one case series), and FASD (one case series). Although psychosis and ADHD were specifically listed in our search strategy, no published trials were identified in children and adolescents.

As far as we are aware, this is the first systematic review of trials of CBP in neuropsychiatric and neurodevelopmental disorders in children and adolescents. There are a few reviews of trials with adult cohorts [27, 121] or mixed child and adult cohorts, however, these focused mostly on adult psychiatric conditions [65, 115, 122]. The most recent review identified substantially more RCTs of CBP in adults (*N* = 31) [27] than we found in children (*N* = 1). In addition to the psychiatric conditions identified in our review, the review with adults identified two RCTs of CBP for treating anorexia nervosa and one RCT for obsessive–compulsive disorder (OCD) in adults. The trials in adults with anorexia nervosa suggest dronabinol may have some benefit but the trial in adults with OCD found smoking cannabis did not improve OCD symptoms [27]. A recent review of studies conducted with children for a range of non-neuropsychiatric health conditions identified eight RCTs but evidence for the efficacy of CBP was only found for treatment of seizures in Dravet syndrome [30].

These existing reviews combined with our current review suggest there is insufficient evidence to recommend prescribing CBP for neuropsychiatric or neurodevelopmental disorders in children and adolescents, and limited evidence for adult cohorts.

### Adverse effects

Consistent with previous reviews, we found that most adverse effects from CBP were mild to moderate, transient, and less severe than those resulting from medications commonly used for psychiatric disorders in paediatric [30, 123] and adult cohorts [27, 65, 115, 121, 122, 124].

In total 44/310 (14%) participants withdrew. Reasons were provided for 38/44 (86%) and included low efficacy (*n* = 17), adverse effects (*n* = 19) and other reasons such as difficulty administering the drug and family circumstances. The most common adverse effects were gastrointestinal symptoms, restlessness, sleepiness, sleeplessness, loss of appetite, weight gain and nervousness (Online Resource 2). The trials we identified had a duration of treatment of 8 weeks (RCT) and 12 weeks (open-label trial), whereas the average duration in RCTs with adult cohorts is 4–6 weeks [121]. Although the existing literature suggests a good safety profile of CBD for short term use, longer trials with consistency in reporting of adverse effects are required to determine whether the benefits of CBD outweigh the adverse effects. More research is needed to confirm the safety profile of THC and other CBP in children.

Adolescence is a time of heightened neurodevelopmental change and there is debate as to whether the adolescent brain is at greater vulnerability to the effects of cannabis [125]. Systematic reviews and meta-analyses suggest an association between recreational cannabis use in adolescence and poorer neurocognitive functioning, most often with heavy use [126, 127]. Most studies have focused on whole plant cannabis, and it is not known whether these effects occur with long-term use of specific cannabinoids, such as CBD. Future RCTs with adolescents should include cannabinoids thought to be least harmful, long-term follow up, and measurement of changes in neurocognitive functioning [127]. Several meta-analyses have also found cannabis use in adolescents is modestly associated with the onset of depression, suicidal ideation, and suicide attempts in early adulthood [128, 129] so these symptoms should be carefully monitored. The trials identified in our review were too short to draw conclusions about the safety of long-term CBP use in childhood and adolescence, highlighting the need for longer trials.

One of the case series identified in the current review reported an increase in “psycho-social behavioural disturbance” in a patient with ASD following administration of a CE [43]. Consistent with this finding, a review of trials conducted with adults from the general population found that acute administration of THC can induce some symptoms of schizophrenia and other psychiatric disorders [130]. A recently published collection of case reports noted that three young men with ASD developed suspected cannabis-induced mania/psychosis. However, two of the three adults had a family history of bipolar disorder and the third, without a family history of bipolar or schizophrenia, had presented with signs of prodromal psychosis a few months before starting a CBP [131]. These reports raise concern but are insufficient to determine whether people with ASD are at an increased risk of cannabis-induced psychosis. Future trials of CBP in ASD cohorts should carefully monitor for signs of bipolar disorder or schizophrenia. The current evidence suggests that although there may be a causal association between CBP and schizophrenia in some cases, other risk factors like genetic predisposition, obstetric complications and adverse childhood experiences also contribute [132].

### Concomitant medication

More severe adverse effects (SAE) of CBD have been reported in RCTs in patients with epilepsy and to a lesser degree psychosis than in the general population or in patients with a range of conditions, including social anxiety disorder [133]. The higher rate of SAEs in epilepsy and psychosis is thought to be due to drug-to-drug interactions. CBD and THC are metabolised by and inhibit cytochrome P450 (CYP) enzymes, which are involved in the metabolism of many common medications [134, 135]. Two medications known to interact with CBD are valproate and clobazam. It is recommended that patients taking valproate and CBD have their liver enzymes monitored regularly, and patients taking clobazam and CBD be closely monitored for signs of somnolence and lethargy [133]. Given the potential for drug-drug interaction, it is recommended that clinicians prescribing CBP with concomitant medications “start low and go slow”.

### Limitations and ways forward

Due to the broad scope of the review, we limited our search to a 40-year period that aligned with the introduction of the DSM-III, although we acknowledge that there was some interest in the medical use of cannabis pre-1980. By not providing an exhaustive list of psychiatric disorders in the search strategy it is possible that we missed articles related to psychiatric conditions such as eating disorders and self-harm, through these were not the target of our review. We also identified some articles that included participants aged both above and below 18 years but had to be excluded because data were not separated by age. Due to the small sample sizes of the two trials, we did not contact authors for individual child data (see Online Resource 4 for a list of these articles). Our review focused on any natural or synthetic cannabinoid product, most of which work on the CB_1_ receptors. Our search strategy did not include other drugs that act on the cannabinoid system and increase the concentration of endogenous endocannabinoid, such as palmitoylethanolamide.

A strength of this review is that it includes the rationale for use of CBP in a range of neurodevelopmental and neuropsychiatric disorders in children and adolescents and provides a summary of the current evidence and trials in progress. The conclusions of the review are limited by the lack of high-quality, primary research and the paucity of RCTs. To minimise risk of bias, well-powered RCTs that use a consistent approach to the type, dose, duration, and mode of administration of CBP are required. The eligible population and diagnostic criteria, the behaviour/symptom to be addressed, and the primary outcome must be clearly defined. Responses to treatment should be measured using validated tools, ideally by an assessor who is blinded to allocation of the intervention. Adverse events must also be systematically recorded and evaluated.

## Conclusion

There is growing societal interest in the efficacy of CBP for a range of neuropsychiatric and neurodevelopmental disorders in children and adolescents. This is likely driven by (i) the frustration of patients and families with the limited efficacy and high rates of adverse effects of existing medications and (ii) the fact that cannabis is considered ‘natural’ and is legalised in some countries, which creates a perception that CBP are ‘safe’. Until good evidence is available to support the efficacy of CBP in neuropsychiatric and neurodevelopmental disorders in children and adolescents, clinicians must balance patients’/parents’ expectations with the available evidence.

### Supplementary Information

Below is the link to the electronic supplementary material.

Supplementary file1 (PDF 225 kb)

Supplementary file2 (XLSX 62 kb)

Supplementary file3 (DOCX 38 kb)

Supplementary file4 (DOCX 23 kb)

Supplementary file5 (PDF 91 kb)
