# Supplementary material for: Efficacy of cannabinoids in neurodevelopmental and neuropsychiatric disorders among children and adolescents: a systematic review
Source: Eur Child Adolesc Psychiatry. 2023 Mar 3;33(2):505–26. doi: 10.1007/s00787-023-02169-w (PMC10869397; doi:10.1007/s00787-023-02169-w)
Supplement: Supplementary file 1 — Supplementary file1 (PDF 225 kb) [file 787_2023_2169_MOESM1_ESM.pdf]

Supplementary Material 1

Article: Efficacy of cannabinoids in neurodevelopmental and neuropsychiatric disorders among children and adolescents: a systematic review

Journal: European Child & Adolescent Psychiatry

Authors: Lauren J. Rice[1,2], Lisa Cannon[1,3], Navin Dadlani[4], Melissa Cheung[1,2], Stewart L. Einfeld[4], Daryl Efron[5,6], David R. Dossetor[2], Elizabeth J. Elliott[1,2]

1. The University of Sydney, Faculty of Medicine and Health, Specialty of Child and Adolescent Health, Sydney, New South Wales, Australia
2. Sydney Children's Hospitals Network, Kids Research
3. Telethon Kids Institute, Perth Children's Hospital, Perth, Western Australia, Australia
4. The University of Sydney, Faculty of Medicine and Health, Brain and Mind Centre, Sydney, New South Wales, Australia
5. Health Services, Murdoch Children's Research Institute, Department of General Paediatrics, Royal Children's Hospital
6. University of Melbourne, Department of Paediatrics

Correspondence: Lauren Rice, lauren.rice@sydney.edu.au

Search strategy for EFFICACY

Search for: limit 21 to (human and yr="1980 -Current" and (preschool child <1 to 6 years> or school child <7 to 12 years> or adolescent <13 to 17 years>))

Results: 1

Embase Classic+Embase <1947 to 2019 October 04>			
**#**	**Search Statement**	**Results**	**Annotation**
1	exp cannabinoid/	66067	
2	(cannabi* or hemp* or marijuana* or nabilone or thc* or sativex or dronabinol or Nabiximol* or epidiolex or Levonantradol or Ajulemic acid or ECP002A or tetrahydrocannabinol).mp.	89434	
3	1 or 2	94006	
4	exp psychosis/	302918	
5	attention deficit disorder/	58411	
6	tic/	9545	
7	Gilles de la Tourette syndrome/	8013	
8	intellectual impairment/	25752	
9	fragile X syndrome/	8314	
10	exp autism/	66143	
11	posttraumatic stress disorder/	57130	
12	exp anxiety disorder/	235210	
13	major depression/	61354	
14	exp bipolar disorder/	63127	
15	fetal alcohol syndrome/	6604	
16	(psychosis or psychoses or psychotic or schizophreni* or adhd or attention deficit disorder*).mp.	366094	
17	(tics or tourette* or intellectual development disorder* or intellectual disabilit* or fragile x).mp.	46441	
18	(autism or autistic or Child Behavio?r Disorder* or pervasive development disorder or major depression or f?etal alcohol or post traumatic stress disorder* or posttraumatic stress disorder*).mp.	209585	
19	(anxiety disorder* or severe behavio?ral disturbance* or challenging behavio?r).mp.	92879	
20	4 or 5 or 6 or 7 or 8 or 9 or 10 or 11 or 12 or 13 or 14 or 15 or 16 or 17 or 18 or 19	786493	
21	3 and 20	13366	
22	limit 21 to (human and yr="1980 -Current" and (preschool child <1 to 6 years> or school child <7 to 12 years> or adolescent <13 to 17 years>))	1907	

Execute Searches in Ovid

Search for: limit 24 to (humans and yr="1980 -Current" and "all child (0 to 18 years)")

Results: 1

Ovid MEDLINE(R) ALL <1946 to October 04, 2019>			
**#**	**Search Statement**	**Results**	**Annotation**
1	exp Cannabinoids/	13338	
2	Cannabis/	8635	
3	(cannabi* or hemp* or marijuana* or nabilone or thc* or sativex or dronabinol or Nabiximol* or epidiolex or Levonantradol or Ajulemic acid or ECP002A or tetrahydrocannabinol).mp.	54594	
4	1 or 2 or 3	54594	
5	psychotic disorders/ or psychoses, substance-induced/	48917	
6	exp Schizophrenia/	102001	
7	exp "Attention Deficit and Disruptive Behavior Disorders"/	31325	
8	Tics/	866	
9	Tourette Syndrome/	4203	
10	exp Intellectual Disability/	93687	
11	Fragile X Syndrome/	4877	
12	exp Child Development Disorders, Pervasive/	32767	
13	bipolar disorder/	39203	
14	Child Behavior Disorders/	20021	
15	Depressive Disorder, Major/	28535	
16	Fetal Alcohol Spectrum Disorders/	4056	
17	Stress Disorders, Post-Traumatic/	30880	
18	exp Anxiety Disorders/	77261	
19	(psychosis or psychoses or psychotic or schizophreni* or adhd or attention deficit disorder*).mp.	231289	
20	(tics or tourette* or intellectual development disorder* or intellectual disabilit* or fragile x).mp.	75871	
21	(autism or autistic or Child Behavio?r Disorder* or pervasive development disorder or major depression or f?etal alcohol or post traumatic stress disorder* or posttraumatic stress disorder*).mp.	122140	
22	(anxiety disorder* or severe behavio?ral disturbance* or challenging behavio?r).mp.	49854	
23	5 or 6 or 7 or 8 or 9 or 10 or 11 or 12 or 13 or 14 or 15 or 16 or 17 or 18 or 19 or 20 or 21 or 22	560264	
24	4 and 23	4874	
25	limit 24 to (humans and yr="1980 -Current" and "all child (0 to 18 years)")	1534	

Execute Searches in Ovid

```
Search for: 24 and 25

Results: 1
```

EBM Reviews - Cochrane Central Register of Controlled Trials <August 2019>			
**#**	**Search Statement**	**Results**	**Annotation**
1	exp cannabinoids/	731	
2	cannabis/	291	
3	(cannabi* or hemp* or marijuana* or nabilone or thc* or sativex or dronabinol or Nabiximol* or epidiolex or Levonantradol or Ajulemic acid or ECP002A or tetrahydrocannabinol).mp.	4554	
4	1 or 2 or 3	4554	
5	exp "Schizophrenia and Disorders with Psychotic Features"/	8104	
6	Tic disorders/	97	
7	tics/	61	
8	Tourette syndrome/	226	
9	exp intellectual disability/	1275	
10	exp Child Development Disorders, Pervasive/	1107	
11	Child behavior disorders/	828	
12	Stress Disorders, Post-Traumatic/	2215	
13	exp anxiety disorders/	7964	
14	exp depression/	10330	
15	exp Depressive Disorder/	10814	
16	exp bipolar disorder/	2399	
17	Fetal Alcohol Spectrum Disorders/	64	
18	(psychosis or psychoses or psychotic or schizophreni* or adhd or attention deficit disorder*).mp.	26645	
19	(tics or tourette* or intellectual development disorder* or intellectual disabilit* or fragile x).mp.	2013	
20	(autism or autistic or Child Behavio?r Disorder* or pervasive development disorder or major depression or f?etal alcohol or post traumatic stress disorder* or posttraumatic stress disorder*).mp.	15983	
21	(anxiety disorder* or severe behavio?ral disturbance* or challenging behavio?r).mp.	9728	
22	5 or 6 or 7 or 8 or 9 or 10 or 11 or 12 or 13 or 14 or 15 or 16 or 17 or 18 or 19 or 20 or 21	67914	
23	4 and 22	715	
24	limit 23 to yr="1980 -Current"	699	
25	adolescent/ or child/ or infant/	130670	
26	24 and 25	112	

Execute Searches in Ovid

Search for: limit 24 to (human and yr="1980 -Current")

Results: 1

PsycINFO <1806 to September Week 5 2019>

#	Search Statement	Results	Annotation
1	exp cannabinoids/	5283	
2	exp cannabis/	7912	
3	(cannabi* or hemp* or marijuana* or nabilone or thc* or sativex or dronabinol or Nabiximol* or epidiolex or Levonantradol or Ajulemic acid or ECP002A or tetrahydrocannabinol).mp.	25547	
4	1 or 2 or 3	25578	
5	exp psychosis/	113480	
6	exp schizophrenia/	88619	
7	exp attention deficit disorder/	26116	
8	tics/	1724	
9	tourette syndrome/	3082	
10	exp intellectual development disorder/	43900	
11	fragile x syndrome/	1733	
12	exp bipolar disorder/	29584	
13	exp autism spectrum disorders/	41401	
14	exp major depression/	125391	
15	fetal alcohol syndrome/	1715	
16	exp posttraumatic stress disorder/	31398	
17	exp anxiety disorders/	52309	
18	(psychosis or psychoses or psychotic or schizophreni* or adhd or attention deficit disorder*).mp.	213457	
19	(tics or tourette* or intellectual development disorder* or intellectual disabilit* or fragile x).mp.	56470	
20	(autism or autistic or pervasive development disorder or major depression or f?etal alcohol or post traumatic stress disorder* or posttraumatic stress disorder*).mp.	215343	
21	(anxiety disorder* or severe behavio?ral disturbance* or challenging behavio?r).mp.	52396	
22	5 or 6 or 7 or 8 or 9 or 10 or 11 or 12 or 13 or 14 or 15 or 16 or 17 or 18 or 19 or 20 or 21	532252	
23	4 and 22	4413	
24	limit 23 to (100 childhood <birth to age 12 yrs> or 200 adolescence <age 13 to 17 yrs>)	970	
25	limit 24 to (human and yr="1980 -Current")	954	

Execute Searches in Ovid

Search strategy for SAFETY

```
Search for: limit 29 to (human and clinical trial and yr="1980 -Current" and (preschool child
<1 to 6 years> or school
child <7 to 12 years> or adolescent <13 to 17 years>))

Results: 1
```

Embase Classic+Embase <1947 to 2019 October 04>			
#	Search Statement	Results	Annotation
1	exp cannabinoid/	66067	
2	(cannabi* or hemp* or marijuana* or nabilone or thc* or sativex or dronabinol or Nabiximol* or epidiolex or Levonantradol or Ajulemic acid or ECP002A or tetrahydrocannabinol).mp.	89434	
3	1 or 2	94006	
4	exp psychosis/	302918	
5	attention deficit disorder/	58411	
6	tic/	9545	
7	Gilles de la Tourette syndrome/	8013	
8	intellectual impairment/	25752	
9	fragile X syndrome/	8314	
10	exp autism/	66143	
11	posttraumatic stress disorder/	57130	
12	exp anxiety disorder/	235210	
13	major depression/	61354	
14	exp bipolar disorder/	63127	
15	fetal alcohol syndrome/	6604	
16	(psychosis or psychoses or psychotic or schizophreni* or adhd or attention deficit disorder*).mp.	366094	
17	(tics or tourette* or intellectual development disorder* or intellectual disabilit* or fragile x).mp.	46441	
18	(autism or autistic or Child Behavio?r Disorder* or pervasive development disorder or major depression or f?etal alcohol or post traumatic stress disorder* or posttraumatic stress disorder*).mp.	209585	
19	(anxiety disorder* or severe behavio?ral disturbance* or challenging behavio?r).mp.	92879	
20	exp epilepsy/	249628	
21	exp seizure/	153756	
22	multiple sclerosis/	127812	
23	chronic pain/	58438	
24	spasticity/	27064	
25	"chemotherapy induced nausea and vomiting"/	3150	
26	chemotherapy induced emesis/	5888	
27	(epilep* or Lennox* or Dravet* or spasticity or cinv or chemotherapy* or chronic pain or multiple sclerosis).mp.	1281790	
28	4 or 5 or 6 or 7 or 8 or 9 or 10 or 11 or 12 or 13 or 14 or 15 or 16 or 17 or 18 or 19 or 20 or 21 or 22 or 23 or 24 or 25 or 26 or 27	2111195	
29	3 and 28	19826	
30	limit 29 to (human and clinical trial and yr="1980 -Current" and (preschool child <1 to 6 years> or school child <7 to 12 years> or adolescent <13 to 17 years>))	85	

Execute Searches in Ovid

Search for: limit 30 to (humans and yr="1980 -Current" and "all child (0 to 18 years)" and clinical trial, all)

Results: 1

Ovid MEDLINE(R) ALL <1946 to October 04, 2019>			
**#**	**Search Statement**	**Results**	**Annotation**
1	exp Cannabinoids/	13338	
2	Cannabis/	8635	
3	(cannabi* or hemp* or marijuana* or nabilone or thc* or sativex or dronabinol or Nabiximol* or epidiolex or Levonantradol or Ajulemic acid or ECP002A or tetrahydrocannabinol).mp.	54594	
4	1 or 2 or 3	54594	
5	psychotic disorders/ or psychoses, substance-induced/	48917	
6	exp Schizophrenia/	102001	
7	exp "Attention Deficit and Disruptive Behavior Disorders"/	31325	
8	Tics/	866	
9	Tourette Syndrome/	4203	
10	exp Intellectual Disability/	93687	
11	Fragile X Syndrome/	4877	
12	exp Child Development Disorders, Pervasive/	32767	
13	bipolar disorder/	39203	
14	Child Behavior Disorders/	20021	
15	Depressive Disorder, Major/	28535	
16	Fetal Alcohol Spectrum Disorders/	4056	
17	Stress Disorders, Post-Traumatic/	30880	
18	exp Anxiety Disorders/	77261	
19	(psychosis or psychoses or psychotic or schizophreni* or adhd or attention deficit disorder*).mp.	231289	
20	(tics or tourette* or intellectual development disorder* or intellectual disabilit* or fragile x).mp.	75871	
21	(autism or autistic or Child Behavio?r Disorder* or pervasive development disorder or major depression or f?etal alcohol or post traumatic stress disorder* or posttraumatic stress disorder*).mp.	122140	
22	(anxiety disorder* or severe behavio?ral disturbance* or challenging behavio?r).mp.	49854	
23	exp Epilepsy/	108189	
24	exp Seizures/	61148	
25	Multiple Sclerosis/	50435	
26	Chronic Pain/	12768	
27	Muscle Spasticity/	8832	
28	(epilep* or Lennox* or Dravet* or spasticity or cinv or chemotherapy* or chronic pain or multiple sclerosis).mp.	709152	
29	5 or 6 or 7 or 8 or 9 or 10 or 11 or 12 or 13 or 14 or 15 or 16 or 17 or 18 or 19 or 20 or 21 or 22 or 23 or 24 or 25 or 26 or 27 or 28	1283072	
30	4 and 29	7510	
31	limit 30 to (humans and yr="1980 -Current" and "all child (0 to 18 years)" and clinical trial, all)	143	

Execute Searches in Ovid

Search for: limit 30 to yr="1980 -Current"

Results: 25

EBM Reviews - Cochrane Central Register of Controlled Trials <August 2019>			
**#**	**Search Statement**	**Results**	**Annotation**
1	exp cannabinoids/	731	
2	cannabis/	291	
3	(cannabi* or hemp* or marijuana* or nabilone or thc* or sativex or dronabinol or Nabiximol* or epidiolex or Levonantradol or Ajulemic acid or ECP002A or tetrahydrocannabinol).mp.	4554	
4	1 or 2 or 3	4554	
5	exp "Schizophrenia and Disorders with Psychotic Features"/	8104	
6	Tic disorders/	97	
7	tics/	61	
8	Tourette syndrome/	226	
9	exp intellectual disability/	1275	
10	exp Child Development Disorders, Pervasive/	1107	
11	Child behavior disorders/	828	
12	Stress Disorders, Post-Traumatic/	2215	
13	exp anxiety disorders/	7964	
14	exp depression/	10330	
15	exp Depressive Disorder/	10814	
16	exp bipolar disorder/	2399	
17	Fetal Alcohol Spectrum Disorders/	64	
18	(psychosis or psychoses or psychotic or schizophreni* or adhd or attention deficit disorder*).mp.	26645	
19	(tics or tourette* or intellectual development disorder* or intellectual disabilit* or fragile x).mp.	2013	
20	(autism or autistic or Child Behavio?r Disorder* or pervasive development disorder or major depression or f?etal alcohol or post traumatic stress disorder* or posttraumatic stress disorder*).mp.	15983	
21	(anxiety disorder* or severe behavio?ral disturbance* or challenging behavio?r).mp.	9728	
22	exp Epilepsy/	2725	
23	muscle spasticity/	785	
24	exp multiple sclerosis/	2991	
25	chronic pain/	1876	
26	(epilep* or Lennox* or Dravet* or spasticity or cinv or chemotherapy* or chronic pain or multiple sclerosis).mp.	99509	
27	5 or 6 or 7 or 8 or 9 or 10 or 11 or 12 or 13 or 14 or 15 or 16 or 17 or 18 or 19 or 20 or 21 or 22 or 23 or 24 or 25 or 26	165923	
28	4 and 27	1258	
29	adolescent/ or child/ or infant/	130670	
30	28 and 29	147	
31	limit 30 to yr="1980 -Current"	142	

Execute Searches in Ovid

```
Search for: limit 29 to (human and "0300 clinical trial" and (childhood  or adolescence <13 to 17 years>) and yr="1980 -Current")

Results: 1
```

PsycINFO <1806 to September Week 5 2019>			
**#**	**Search Statement**	**Results**	**Annotation**
1	exp cannabinoids/	5283	
2	exp cannabis/	7912	
3	(cannabi* or hemp* or marijuana* or nabilone or thc* or sativex or dronabinol or Nabiximol* or epidiolex or Levonantradol or Ajulemic acid or ECP002A or tetrahydrocannabinol).mp.	25547	
4	1 or 2 or 3	25578	
5	exp psychosis/	113480	
6	exp schizophrenia/	88619	
7	exp attention deficit disorder/	26116	
8	tics/	1724	
9	tourette syndrome/	3082	
10	exp intellectual development disorder/	43900	
11	fragile x syndrome/	1733	
12	exp bipolar disorder/	29584	
13	exp autism spectrum disorders/	41401	
14	exp major depression/	125391	
15	fetal alcohol syndrome/	1715	
16	exp posttraumatic stress disorder/	31398	
17	exp anxiety disorders/	52309	
18	(psychosis or psychoses or psychotic or schizophreni* or adhd or attention deficit disorder*).mp.	213457	
19	(tics or tourette* or intellectual development disorder* or intellectual disabilit* or fragile x).mp.	56470	
20	(autism or autistic or pervasive development disorder or major depression or f?etal alcohol or post traumatic stress disorder* or posttraumatic stress disorder*).mp.	215343	
21	(anxiety disorder* or severe behavio?ral disturbance* or challenging behavio?r).mp.	52396	
22	exp epilepsy/	26722	
23	exp seizures/	15796	
24	multiple sclerosis/	12431	
25	chronic pain/	12873	
26	chemotherapy/	2925	
27	(epilep* or Lennox* or Dravet* or spasticity or cinv or chemotherapy* or chronic pain or multiple sclerosis).mp.	85323	
28	5 or 6 or 7 or 8 or 9 or 10 or 11 or 12 or 13 or 14 or 15 or 16 or 17 or 18 or 19 or 20 or 21 or 22 or 23 or 24 or 25 or 26 or 27	608284	
29	4 and 28	5177	
30	limit 29 to (human and "0300 clinical trial" and (childhood <birth to 12 years> or adolescence <13 to 17 years>) and yr="1980 -Current")	24	

Execute Searches in Ovid
