# Supplementary material for: Efficacy of cannabinoids in neurodevelopmental and neuropsychiatric disorders among children and adolescents: a systematic review
Source: Eur Child Adolesc Psychiatry. 2023 Mar 3;33(2):505–26. doi: 10.1007/s00787-023-02169-w (PMC10869397; doi:10.1007/s00787-023-02169-w)
Supplement: Supplementary file 3 — Supplementary file3 (DOCX 38 kb) [file 787_2023_2169_MOESM3_ESM.docx]

**Supplementary Material 3**

| Article: | Efficacy of cannabinoids in neurodevelopmental and neuropsychiatric disorders among children and adolescents: a systematic review |
| --- | --- |
| Journal: | European Child & Adolescent Psychiatry |
| Authors: | Lauren J. Rice^1,2^, Lisa Cannon^1,3^, Navin Dadlani^4^, Melissa Cheung^1,2^, Stewart L. Einfeld^4^, Daryl Efron^5,6^, David R. Dossetor^2^, Elizabeth J. Elliott^1,2^ |

1. The University of Sydney, Faculty of Medicine and Health, Specialty of Child and Adolescent Health, Sydney, New South Wales, Australia
2. Sydney Children’s Hospitals Network, Kids Research
3. Telethon Kids Institute, Perth Children’s Hospital, Perth, Western Australia, Australia
4. The University of Sydney, Faculty of Medicine and Health, Brain and Mind Centre, Sydney, New South Wales, Australia
5. Health Services, Murdoch Children’s Research Institute, Department of General Paediatrics, Royal Children's Hospital
6. University of Melbourne, Department of Paediatrics

Correspondence: Lauren Rice, lauren.rice@sydney.edu.au

Summary and Quality of Evidence of Case Series and Case Reports

**Autism Spectrum Disorder (ASD)**

Delta-9-tetrahydrocannabinol (THC)

A case report of a six-year-old male who received THC (daily dose 3.62mg) over six months reported significant improvements in hyperactivity, lethargy, irritability, and inappropriate speech. No adverse effects were observed [1].

Whole plant or CBD and THC

The case report included a 15-year-old male with ASD treated with the cannabidiol-based extract (CBE) (0.001% THC and 0.02% CBD) [2]. Improvements were seen in anxiety, aggression, irritability, socialization, communication, and sleep and were still evident 2 years after initiation. No adverse effects were reported, and an attempt at weaning off CBE increased irritability and aggressiveness. While partial responses were seen at the initial dose of 0.1 mL twice a day, responses were improved at a dose of 0.2 mL twice a day - suggesting possible benefits of a relatively low dose of CBE [2].

**Fragile X Syndrome (FXS)**

The case report was part of a case series (n=3), however, only one of the participants was under the age of 19 and eligible for our review. This participant was a 3.5-year-old male who was administered 50mg of CBD per day via an oral CBD paste (18%- 23.5% CBD, 0.03% THC) since 15-months of age [3]. Whilst on CBD treatment parents reported improvements in a range of parameters, such as feeding, weight gain, oral-motor coordination, motor skills, vocalisation and language skills, willingness to explore new places, a decrease in self-stimulatory behaviour, social avoidance, sensory sensitivities, and hyperactivity. Discontinuation of CBD (at 30 months of age) led to re-emergence of prior symptoms (i.e., anxiety, meltdowns, sleep difficulties), which reduced when CBD treatment was reinitiated at 3 years of age. According to the Vineland Adaptive Behaviour Scale (VABS), he had also maintained his score from 1 to 3 years of age, indicating an improvement (individuals with FXS tend to have a decrease in adaptive function scores over this time). No adverse events were observed [3].

*Quality of evidence*

In the case report, adaptive behaviour and weight were the only outcomes formally measured before and after CBD treatment and not all adaptive behaviour subscales improved with treatment. All other outcome variables were based on parent and clinician observations. The participant in the case study began speech and occupational therapy one month after beginning CBD and sertraline four months after beginning CBD, which may have contributed to the improvements reported.

**Mood Disorders**

A case series (n=5), which included one individual aged 18 years or younger [4], and a case report [5] suggest marijuana/CBD capsules may improve depressive symptoms in adolescents. Both cases were of 16-year-old males, one who began using marijuana several months after his onset of depressive symptoms in eighth grade, was hospitalised for major depression (first episode) and had a history of dysthymia and ADHD [4]. He self-reported (retrospectively) that his depressive symptoms increased when he was unable to use marijuana; and that marijuana produced a full relief of his symptoms, while fluoxetine only provided partial relief.

The other case had several symptoms in addition to severe depression (including paranoia, derealization, attention deficit, social anxiety and social withdrawal, and multiple substance abuse). He was administered CBD capsules for over 6 months (increased from 50mg twice a day to 300mg twice a day over the first three weeks) [5]. Over this time, his depressive and anxiety symptoms gradually improved, his risk of psychosis reduced, and he reported no adverse events. After the first 3 weeks of CBD treatment, he requested to cease his antidepressant medication (Sertraline), which led to no difference in mood or anxiety symptoms. He also ceased abusing drugs, including THC, with no observed withdrawal symptoms; however, his improvement in paranoid and dissociative symptoms may be attributed to the cessation of THC [5].

*Quality of Evidence*

The case series included only one participant aged 18 years or younger and only briefly mentioned the patient’s medical history. The authors did not formally administer and control the marijuana dose or assess the patient’s symptoms. Rather, the report is based on the patient self-medicating and self-reporting improvements. It is unclear whether the reported improvement in mood extended beyond the temporary euphoric effects of acute intoxication. Finally, the patient met DMS-IV criteria for cannabis or polysubstance dependence (the authors do not specify) and therefore his report in mood changes might have been confounded by his drug-seeking behaviour [4].

The young man in the second case report had several co-occurring mental illnesses, including multiple substance abuse so, like the above patient, self-reports of improved mood could be confounded by his drug-seeking behaviour. He also received talk therapy during treatment with CBD, which may have contributed to the improvement in mood. The quality of this case report was better than the first: CBD was formally administered and controlled, and the authors used validated assessments pre- and post-administration. However, more information was needed about the patient’s history.

**Anxiety Disorders**

The 14-year-old girl with specific phobia (i.e., medical procedures, receiving injections, seeing blood, animals) and social phobia was administered CBD capsules for 26 weeks (dosage 100mg/d to 600mg over 19 weeks). Although the case still met DSM-IV diagnostic criteria for both phobias following treatment, there was a reduction in symptom expression, and a decrease on the Clinical Global Impression Severity Scale (CGI-S), suggesting improvement in symptoms but not remission of the phobias [6]. Adverse effects were not mentioned.

*Quality of evidence*

The patient described in the case report had additional health problems (Crohn’s disease) and was self-medicating with her source of CBD before moving to the product provided by the authors and it’s unclear whether there was a washout period between products.

**Post-Traumatic Stress Disorder (PTSD)**

Two cases reported in the same article (11-year-old female, and 3-year-10-month-old male) were orally administered Dronabinol (THC) after suffering physical trauma as a result of traffic accidents, which according to parental and clinical reports resulted in improved PTSD reactions (e.g. ate more, more accessible/open to therapy, was happier) [7]. However, when given a higher dose the 11-year-old demonstrated verbal inhibition (sexual content) and associative thinking. The 11-year-old discontinued treatment after three months, with no signs of withdrawal, while mood improved, and weight remained stable.

The other two cases were administered CBD as either CBD oil over 5 months (10-year-old female) [8] or CBD capsules (CBD powder with 99.9% purity dissolved in corn oil) over 7 days (15-year-old female) [9], for reducing anxiety and insomnia due to sexual abuse. For the 10-year-old, based on monthly measures of sleep (Sleep disturbance scale for children) and anxiety (screen for anxiety-related disorders (SCARED)), there was a steady reduction in anxiety and insomnia scores. The case was routinely asked about adverse events (e.g., headaches) but did not report any [8]. For the 15-year-old, CBD did not prevent an increase in anxiety after she recollected the traumatic event two days after the event or prevent the development of PTSD. However, anxiety also did not increase when she was re-exposed to the account sixteen days after the event, suggesting a possible interference of CBD [9]. The case report provided no information on adverse effects [9].

*Quality of evidence/risk of bias*

The quality was poor for the two cases that were administered THC. These reports did not properly describe the patient’s history, current clinical condition, diagnostic methods, or the intervention [7]. The two case reports that described patients who were administered CBD were of a higher quality. The authors adequately described the intervention and used validated pre-and post-intervention measures [9]. However, the results must still be interpreted with caution as there is a high risk of bias given the raters were not blind to the intervention and there was no control group.

**Tourette’s syndrome (TS)**

The first case series included one child, a 16-year-old male given vaporized dronabinol (initially 16.8mg/day for 8 months) [10]. Based on self-report and clinical observation, participants had significant improvements in motor tics (e.g., head nodding), vocal tics (e.g., speech fluency), comorbid conditions and quality of life (e.g., social contact, school performance, impulsive behaviour, felt calmer). At 1 year follow-up, the 16-year-old relapsed due to noncompliance and worsening symptoms. He reported improvements (e.g. overall tics and speech fluency) after restarting treatment (22.4-33.6mg/day for 8 months) [10]. The other case series also included a 16-year-old male who smoked marijuana occasionally (1-2 cigarettes/week) and an 18-year-old male, who smoked marijuana regularly (5-6 cigarettes/week), both had ADHD. They were retrospectively interviewed regarding the efficacy of marijuana use and both reported a reduction of motor tics and vocal tics. The 18-year-old had been smoking for more than 3 years, with no decrease in efficacy and was not being treated with other medication [11]. The 16-year-old was treated with a neuroleptic. Regarding all three studies, no serious adverse events were reported, with only one case reporting mild transient events after initial intake (15-year-old) [12].

For the case report, THC was administered over 9 weeks as an ‘add-on’ treatment for a 15-year-old male with TS and ADHD [12]. Based on 5 measures (Yale Global Tic Severity Scale; Conners’ Teacher Rating Scale; the Gilles de la Tourette Syndrome- Quality of Life Scale), the participant's tic severity reduced, and quality of life improved between baseline and week 7. Although there was a slight decrease in ADHD associated symptoms after week 8, methylphenidate was added to his treatment to further decrease ADHD symptoms, resulting in no increase in tics or inner tension [12].

*Quality of evidence/risk of bias*

In the case report, THC was administered as an add on to aripiprazole and risperidone, both of which were kept constant throughout the THC trial. Validated pre- and post-measures were used to assess changes at baseline and after 7 weeks of THC before methylphenidate was added to treat ADHD symptoms [12]. Overall, this case report was well described. The case series with only one participant under 18 years of age reported improvements in tics and behaviour problems. However, these improvements were based on clinical evaluation and subjective reporting by the participant. No formal pre- and post-measures were used to assess change {Jakubovski, 2017 #162}. The second case series included two participants within the age range. In this study, the authors retrospectively interviewed people with TS about their marijuana use, which means the drug was self-administered and varied in both dose and strain {Müller-Vahl, 1998 #169}. There was also no pre-and post-assessments, rather efficacy was based on a retrospective interview that was presumably developed for the study. Overall, these case reports and series offer limited evidence of the efficacy of CBP for treating symptoms of paediatric TS.

**Fetal Alcohol Spectrum Disorder (FASD)**

The case series (n=5) [13] included 2 children (a 5-year 4-month-old male; and a 12-year-old male) with FASD and disruptive behaviour. Both received CBD oil (20% CBD, 0.2% THC: and 15% CBD, 1% THC, respectively). Using a visual analogue scale (VAS) for evaluating disruptive symptoms (the Nisonger Child Behavior Rating Form), parents reported behavioural change. In the 5-year-old a reduction in aggression towards other children, tantrums and disappearances was reported as were improvements in communicating feelings and asking for permission. In the 12-year-old reductions in aggression, restlessness and impulsivity were reported. Neither case experienced any adverse events [13].

References

1. Kurz R, Blaas K (2010) Use of dronabinol (delta-9-THC) in autism: a prospective single-case-study with an early infantile autistic child. Cannabinoids 5:4-6

2. Ponton JA, Smyth K, Soumbasis E, Llanos SA, Lewis M, Meerholz WA, Tanguay RL (2020) A pediatric patient with autism spectrum disorder and epilepsy using cannabinoid extracts as complementary therapy: a case report. Journal of Medical Case Reports 14:162-162

3. Tartaglia N, Bonn-Miller M, Hagerman R (2019) Treatment of Fragile X Syndrome with Cannabidiol: A Case Series Study and Brief Review of the Literature. Cannabis and Cannabinoid Research 4:3-9

4. Gruber AJ, Pope HG, Jr., Brown ME (1996) Do patients use marijuana as an antidepressant? Depression 4:77-80

5. Laczkovics C, Kothgassner OD, Felnhofer A, Klier CM (2021) Cannabidiol treatment in an adolescent with multiple substance abuse, social anxiety and depression. Neuropsychiatrie 35:31-34

6. Klier CM, de Gier C, Felnhofer A, Laczkovics C, Amminger PG (2020) A Case Report of Cannabidiol Treatment of a Crohn's Disease Patient With Anxiety Disorder. Journal of Clinical Psychopharmacology 40:90-92

7. Lorenz R (2004) On the application of cannabis in paediatrics and epileptology. Neuro Endocrinology Letters 25:40-44

8. Shannon S, Opila-Lehman J (2016) Effectiveness of Cannabidiol Oil for Pediatric Anxiety and Insomnia as Part of Posttraumatic Stress Disorder: A Case Report. Permanente Journal 20:16-005

9. Bolsoni LM, da Silva TDA, Quintana SM, de Castro M, Crippa JA, Zuardi AW (2019) Changes in Cortisol Awakening Response Before and After Development of Posttraumatic Stress Disorder, Which Cannot be Avoided with Use of Cannabidiol: A Case Report. The Permanente Journal 23

10. Jakubovski E, Muller-Vahl K (2017) Speechlessness in Gilles de la Tourette Syndrome: Cannabis-based medicines improve severe vocal blocking tics in two patients. International Journal of Molecular Sciences 18:1739

11. Müller-Vahl KR, Kolbe H, Schneider U, Emrich HM (1998) Cannabinoids: possible role in patho-physiology and therapy of Gilles de la Tourette syndrome. Acta Psychiatrica Scandinavica 98:502-506

12. Hasan A, Rothenberger A, Munchau A, Wobrock T, Falkai P, Roessner V (2010) Oral DELTA9-tetrahydrocannabinol improved refractory Gilles de la Tourette syndrome in an adolescent by increasing intracortical inhibition: A case report. Journal of Clinical Psychopharmacology 30:190-192

13. Koren G, Cohen R, Sachs O (2021) Use of cannabis in fetal alcohol spectrum disorder. Cannabis and Cannabinoid Research 6:74–76
