# Supplementary material for: Efficacy of cannabinoids in neurodevelopmental and neuropsychiatric disorders among children and adolescents: a systematic review
Source: Eur Child Adolesc Psychiatry. 2023 Mar 3;33(2):505–26. doi: 10.1007/s00787-023-02169-w (PMC10869397; doi:10.1007/s00787-023-02169-w)
Supplement: Supplementary file 4 — Supplementary file4 (DOCX 23 kb) [file 787_2023_2169_MOESM4_ESM.docx]

**Supplementary Material 4**

| Article: | Efficacy of cannabinoids in neurodevelopmental and neuropsychiatric disorders among children and adolescents: a systematic review |
| --- | --- |
| Journal: | European Child & Adolescent Psychiatry |
| Authors: | Lauren J. Rice^1,2^, Lisa Cannon^1,3^, Navin Dadlani^4^, Melissa Cheung^1,2^, Stewart L. Einfeld^4^, Daryl Efron^5,6^, David R. Dossetor^2^, Elizabeth J. Elliott^1,2^ |

1. The University of Sydney, Faculty of Medicine and Health, Specialty of Child and Adolescent Health, Sydney, New South Wales, Australia
2. Sydney Children’s Hospitals Network, Kids Research
3. Telethon Kids Institute, Perth Children’s Hospital, Perth, Western Australia, Australia
4. The University of Sydney, Faculty of Medicine and Health, Brain and Mind Centre, Sydney, New South Wales, Australia
5. Health Services, Murdoch Children’s Research Institute, Department of General Paediatrics, Royal Children's Hospital
6. University of Melbourne, Department of Paediatrics

Correspondence: Lauren Rice, lauren.rice@sydney.edu.au

A list of the publications that were excluded after full-text review because the results of participants within and outside age criteria could not be separated

Aran A, Harel M, Cassuto H, Polyansky L, Schnapp A, Wattad N, Shmueli D, Golan D, Castellanos FX (2021). Cannabinoid treatment for autism: a proof-of-concept randomized trial. Molecular Autism, 12(1), pp.6.

Barchel D, Stolar O, De-Haan T, Ziv-Baran T, Saban N, Fuchs DO, Koren G and Berkovitch M (2019). Oral Cannabidiol Use in Children With Autism Spectrum Disorder to Treat Related Symptoms and Co-morbidities. Front. Pharmacol. 9, pp.1521. doi: 10.3389/fphar.2018.01521.

Grotenhermen, F.: Schnelle, M. (2003). Survey on the medical use of Cannabis and THC in Germany. Journal of Cannabis Therapeutics, 3(2), pp17-40.

Hanna, R.: Shalvoy, A.: Ghose, S.: Cullum, M.: Hill, S.: Sweeney, J.: Keshavan, M.: Pearlson, G.: Clements, B.: Gershon, E.: Tamminga, C. (2016). Cognitive Function in Individuals With Psychosis: Moderation by Adolescent Cannabis Use, Schizophrenia Bulletin, 42(6), pp.1496-1503.

Lake S, Kerr T, Buxton J, Walsh Z, Marshall BD, Wood E, Milloy MJ (2020). Does cannabis use modify the effect of post-traumatic stress disorder on severe depression and suicidal ideation? Evidence from a population-based cross-sectional study of Canadians. Journal of psychopharmacology (Oxford, England) 34:181-188.

Muller-Vahl, K. R.: Kolbe, H.: Dengler, R. (1997). Gilles de la Tourette syndrome. Influence of nicotine, alcohol, and marijuana on the clinical symptome. [German], 68(12), p.985.

Sexton, M.: Cuttler, C.: Finnell, J. S.: Mischley, L. K. (2016). A Cross-Sectional Survey of Medical Cannabis Users: Patterns of Use and Perceived Efficacy. Cannabis and Cannabinoid Research, 1(1) pp. 131-138

Turna, J.: Simpson, W.: Patterson, B.: Lucas, P.: Van Ameringen, M. (2019). Cannabis use behaviors and prevalence of anxiety and depressive symptoms in a cohort of Canadian medicinal cannabis users, Journal of Psychiatric Research, 111. pp. 134-139

Usdin, M.: Mesnage, V.: Gonce, M.: Jedynak, C. P. (2005). Gilles de la Tourette syndrome: A self-administered assessment questionnaire. [French]. Revue Neurologique 161(8-9), p.795

Vazquez-Bourgon J, Ortiz-Garcia de la Foz V, Suarez-Pereira I, Iruzubieta P, Arias-Loste MT, Setien-Suero E, Ayesa-Arriola R, Gomez-Revuelta M, Crespo J, Facorro BC (2019) Cannabis consumption and non-alcoholic fatty liver disease. A three years longitudinal study in first episode non-affective psychosis patients. Progress in Neuro-Psychopharmacology & Biological Psychiatry 95

Wan, B. A., M.Phil, DeAngelis, C., PharmD., Lam, H., M.L.S., Ganesh, V., B.Sc(C.), Malek, L., B.Sc, Chow, E., M.B.B.S., . . . Shaw, E., M.D. (2017). Efficacy of different varieties of medical cannabis in relieving symptoms. Journal of Pain Management, 10(4), 375-383.
