# Supplementary material for: Efficacy of cannabinoids in neurodevelopmental and neuropsychiatric disorders among children and adolescents: a systematic review
Source: Eur Child Adolesc Psychiatry. 2023 Mar 3;33(2):505–26. doi: 10.1007/s00787-023-02169-w (PMC10869397; doi:10.1007/s00787-023-02169-w)
Supplement: Supplementary file 5 — Supplementary file5 (PDF 91 kb) [file 787_2023_2169_MOESM5_ESM.pdf]

Search strategy for EFFICACY

Search for: limit 21 to (human and yr="1980 -Current" and (preschool child <1 to 6 years> or school child <7 to 12 years> or adolescent <13 to 17 years>))

Results: 1

Embase Classic+Embase <1947 to 2019 October 04>			
#	**Search Statement**	**Results**	**Annotation**
1	exp cannabinoid/	66067	
2	(cannabi* or hemp* or marijuana* or nabilone or thc* or sativex or dronabinol or Nabiximol* or epidiolex or Levonantradol or Ajulemic acid or ECP002A or tetrahydrocannabinol).mp.	89434	
3	1 or 2	94006	
4	exp psychosis/	302918	
5	attention deficit disorder/	58411	
6	tic/	9545	
7	Gilles de la Tourette syndrome/	8013	
8	intellectual impairment/	25752	
9	fragile X syndrome/	8314	
10	exp autism/	66143	
11	posttraumatic stress disorder/	57130	
12	exp anxiety disorder/	235210	
13	major depression/	61354	
14	exp bipolar disorder/	63127	
15	fetal alcohol syndrome/	6604	
16	(psychosis or psychoses or psychotic or schizophreni* or adhd or attention deficit disorder*).mp.	366094	
17	(tics or tourette* or intellectual development disorder* or intellectual disabilit* or fragile x).mp.	46441	
18	(autism or autistic or Child Behavio?r Disorder* or pervasive development disorder or major depression or f?etal alcohol or post traumatic stress disorder* or posttraumatic stress disorder*).mp.	209585	
19	(anxiety disorder* or severe behavio?ral disturbance* or challenging behavio?r).mp.	92879	
20	4 or 5 or 6 or 7 or 8 or 9 or 10 or 11 or 12 or 13 or 14 or 15 or 16 or 17 or 18 or 19	786493	
21	3 and 20	13366	
22	limit 21 to (human and yr="1980 -Current" and (preschool child <1 to 6 years> or school child <7 to 12 years> or adolescent <13 to 17 years>))	1907	

Execute Searches in Ovid

```
Search for: limit 24 to (humans and yr="1980 -Current" and "all child (0 to
18 years)")

Results: 1
```

Ovid MEDLINE(R) ALL <1946 to October 04, 2019>			
**#**	**Search Statement**	**Results**	**Annotation**
1	exp Cannabinoids/	13338	
2	Cannabis/	8635	
3	(cannabi* or hemp* or marijuana* or nabilone or thc* or sativex or dronabinol or Nabiximol* or epidiolex or Levonantradol or Ajulemic acid or ECP002A or tetrahydrocannabinol).mp.	54594	
4	1 or 2 or 3	54594	
5	psychotic disorders/ or psychoses, substance-induced/	48917	
6	exp Schizophrenia/	102001	
7	exp "Attention Deficit and Disruptive Behavior Disorders"/	31325	
8	Tics/	866	
9	Tourette Syndrome/	4203	
10	exp Intellectual Disability/	93687	
11	Fragile X Syndrome/	4877	
12	exp Child Development Disorders, Pervasive/	32767	
13	bipolar disorder/	39203	
14	Child Behavior Disorders/	20021	
15	Depressive Disorder, Major/	28535	
16	Fetal Alcohol Spectrum Disorders/	4056	
17	Stress Disorders, Post-Traumatic/	30880	
18	exp Anxiety Disorders/	77261	
19	(psychosis or psychoses or psychotic or schizophreni* or adhd or attention deficit disorder*).mp.	231289	
20	(tics or tourette* or intellectual development disorder* or intellectual disabilit* or fragile x).mp.	75871	
21	(autism or autistic or Child Behavio?r Disorder* or pervasive development disorder or major depression or f?etal alcohol or post traumatic stress disorder* or posttraumatic stress disorder*).mp.	122140	
22	(anxiety disorder* or severe behavio?ral disturbance* or challenging behavio?r).mp.	49854	
23	5 or 6 or 7 or 8 or 9 or 10 or 11 or 12 or 13 or 14 or 15 or 16 or 17 or 18 or 19 or 20 or 21 or 22	560264	
24	4 and 23	4874	
25	limit 24 to (humans and yr="1980 -Current" and "all child (0 to 18 years)")	1534	

Execute Searches in Ovid

Search for: 24 and 25

Results: 1

EBM Reviews - Cochrane Central Register of Controlled Trials <August 2019>			
**#**	**Search Statement**	**Results**	**Annotation**
1	exp cannabinoids/	731	
2	cannabis/	291	
3	(cannabi* or hemp* or marijuana* or nabilone or thc* or sativex or dronabinol or Nabiximol* or epidiolex or Levonantradol or Ajulemic acid or ECP002A or tetrahydrocannabinol).mp.	4554	
4	1 or 2 or 3	4554	
5	exp "Schizophrenia and Disorders with Psychotic Features"/	8104	
6	Tic disorders/	97	
7	tics/	61	
8	Tourette syndrome/	226	
9	exp intellectual disability/	1275	
10	exp Child Development Disorders, Pervasive/	1107	
11	Child behavior disorders/	828	
12	Stress Disorders, Post-Traumatic/	2215	
13	exp anxiety disorders/	7964	
14	exp depression/	10330	
15	exp Depressive Disorder/	10814	
16	exp bipolar disorder/	2399	
17	Fetal Alcohol Spectrum Disorders/	64	
18	(psychosis or psychoses or psychotic or schizophreni* or adhd or attention deficit disorder*).mp.	26645	
19	(tics or tourette* or intellectual development disorder* or intellectual disabilit* or fragile x).mp.	2013	
20	(autism or autistic or Child Behavio?r Disorder* or pervasive development disorder or major depression or f?etal alcohol or post traumatic stress disorder* or posttraumatic stress disorder*).mp.	15983	
21	(anxiety disorder* or severe behavio?ral disturbance* or challenging behavio?r).mp.	9728	
22	5 or 6 or 7 or 8 or 9 or 10 or 11 or 12 or 13 or 14 or 15 or 16 or 17 or 18 or 19 or 20 or 21	67914	
23	4 and 22	715	
24	limit 23 to yr="1980 -Current"	699	
25	adolescent/ or child/ or infant/	130670	
26	24 and 25	112	

Execute Searches in Ovid

Search for: limit 24 to (human and yr="1980 -Current")

Results: 1

PsycINFO <1806 to September Week 5 2019>			
#	**Search Statement**	**Results**	**Annotation**
1	exp cannabinoids/	5283	
2	exp cannabis/	7912	
3	(cannabi* or hemp* or marijuana* or nabilone or thc* or sativex or dronabinol or Nabiximol* or epidiolex or Levonantradol or Ajulemic acid or ECP002A or tetrahydrocannabinol).mp.	25547	
4	1 or 2 or 3	25578	
5	exp psychosis/	113480	
6	exp schizophrenia/	88619	
7	exp attention deficit disorder/	26116	
8	tics/	1724	
9	tourette syndrome/	3082	
10	exp intellectual development disorder/	43900	
11	fragile x syndrome/	1733	
12	exp bipolar disorder/	29584	
13	exp autism spectrum disorders/	41401	
14	exp major depression/	125391	
15	fetal alcohol syndrome/	1715	
16	exp posttraumatic stress disorder/	31398	
17	exp anxiety disorders/	52309	
18	(psychosis or psychoses or psychotic or schizophreni* or adhd or attention deficit disorder*).mp.	213457	
19	(tics or tourette* or intellectual development disorder* or intellectual disabilit* or fragile x).mp.	56470	
20	(autism or autistic or pervasive development disorder or major depression or f?etal alcohol or post traumatic stress disorder* or posttraumatic stress disorder*).mp.	215343	
21	(anxiety disorder* or severe behavio?ral disturbance* or challenging behavio?r).mp.	52396	
22	5 or 6 or 7 or 8 or 9 or 10 or 11 or 12 or 13 or 14 or 15 or 16 or 17 or 18 or 19 or 20 or 21	532252	
23	4 and 22	4413	
24	limit 23 to (100 childhood <birth to age 12 yrs> or 200 adolescence <age 13 to 17 yrs>)	970	
25	limit 24 to (human and yr="1980 -Current")	954	

Execute Searches in Ovid

Search strategy for SAFETY

```
Search for: limit 29 to (human and clinical trial and yr="1980 -Current" and (preschool child
<1 to 6 years> or school
child <7 to 12 years> or adolescent <13 to 17 years>))

Results: 1
```

Embase Classic+Embase <1947 to 2019 October 04>			
#	Search Statement	Results	Annotation
1	exp cannabinoid/	66067	
2	(cannabi* or hemp* or marijuana* or nabilone or thc* or sativex or dronabinol or Nabiximol* or epidiolex or Levonantradol or Ajulemic acid or ECP002A or tetrahydrocannabinol).mp.	89434	
3	1 or 2	94006	
4	exp psychosis/	302918	
5	attention deficit disorder/	58411	
6	tic/	9545	
7	Gilles de la Tourette syndrome/	8013	
8	intellectual impairment/	25752	
9	fragile X syndrome/	8314	
10	exp autism/	66143	
11	posttraumatic stress disorder/	57130	
12	exp anxiety disorder/	235210	
13	major depression/	61354	
14	exp bipolar disorder/	63127	
15	fetal alcohol syndrome/	6604	
16	(psychosis or psychoses or psychotic or schizophreni* or adhd or attention deficit disorder*).mp.	366094	
17	(tics or tourette* or intellectual development disorder* or intellectual disabilit* or fragile x).mp.	46441	
18	(autism or autistic or Child Behavio?r Disorder* or pervasive development disorder or major depression or f?etal alcohol or post traumatic stress disorder* or posttraumatic stress disorder*).mp.	209585	
19	(anxiety disorder* or severe behavio?ral disturbance* or challenging behavio?r).mp.	92879	
20	exp epilepsy/	249628	
21	exp seizure/	153756	
22	multiple sclerosis/	127812	
23	chronic pain/	58438	
24	spasticity/	27064	
25	"chemotherapy induced nausea and vomiting"/	3150	
26	chemotherapy induced emesis/	5888	
27	(epilep* or Lennox* or Dravet* or spasticity or cinv or chemotherapy* or chronic pain or multiple sclerosis).mp.	1281790	
28	4 or 5 or 6 or 7 or 8 or 9 or 10 or 11 or 12 or 13 or 14 or 15 or 16 or 17 or 18 or 19 or 20 or 21 or 22 or 23 or 24 or 25 or 26 or 27	2111195	
29	3 and 28	19826	
30	limit 29 to (human and clinical trial and yr="1980 -Current" and (preschool child <1 to 6 years> or school child <7 to 12 years> or adolescent <13 to 17 years>))	85	

Execute Searches in Ovid

```
Search for: limit 30 to (humans and yr="1980 -Current" and "all child (0 to 18 years)" and
clinical trial, all)

Results: 1
```

Ovid MEDLINE(R) ALL <1946 to October 04, 2019>			
**#**	**Search Statement**	**Results**	**Annotation**
1	exp Cannabinoids/	13338	
2	Cannabis/	8635	
3	(cannabi* or hemp* or marijuana* or nabilone or thc* or sativex or dronabinol or Nabiximol* or epidiolex or Levonantradol or Ajulemic acid or ECP002A or tetrahydrocannabinol).mp.	54594	
4	1 or 2 or 3	54594	
5	psychotic disorders/ or psychoses, substance-induced/	48917	
6	exp Schizophrenia/	102001	
7	exp "Attention Deficit and Disruptive Behavior Disorders"/	31325	
8	Tics/	866	
9	Tourette Syndrome/	4203	
10	exp Intellectual Disability/	93687	
11	Fragile X Syndrome/	4877	
12	exp Child Development Disorders, Pervasive/	32767	
13	bipolar disorder/	39203	
14	Child Behavior Disorders/	20021	
15	Depressive Disorder, Major/	28535	
16	Fetal Alcohol Spectrum Disorders/	4056	
17	Stress Disorders, Post-Traumatic/	30880	
18	exp Anxiety Disorders/	77261	
19	(psychosis or psychoses or psychotic or schizophreni* or adhd or attention deficit disorder*).mp.	231289	
20	(tics or tourette* or intellectual development disorder* or intellectual disabilit* or fragile x).mp.	75871	
21	(autism or autistic or Child Behavio?r Disorder* or pervasive development disorder or major depression or f?etal alcohol or post traumatic stress disorder* or posttraumatic stress disorder*).mp.	122140	
22	(anxiety disorder* or severe behavio?ral disturbance* or challenging behavio?r).mp.	49854	
23	exp Epilepsy/	108189	
24	exp Seizures/	61148	
25	Multiple Sclerosis/	50435	
26	Chronic Pain/	12768	
27	Muscle Spasticity/	8832	
28	(epilep* or Lennox* or Dravet* or spasticity or cinv or chemotherapy* or chronic pain or multiple sclerosis).mp.	709152	
29	5 or 6 or 7 or 8 or 9 or 10 or 11 or 12 or 13 or 14 or 15 or 16 or 17 or 18 or 19 or 20 or 21 or 22 or 23 or 24 or 25 or 26 or 27 or 28	1283072	
30	4 and 29	7510	
31	limit 30 to (humans and yr="1980 -Current" and "all child (0 to 18 years)" and clinical trial, all)	143	

Execute Searches in Ovid

```
Search for: limit 30 to yr="1980 -Current"

Results: 25
```

EBM Reviews - Cochrane Central Register of Controlled Trials <August 2019>			
#	**Search Statement**	**Results**	**Annotation**
1	exp cannabinoids/	731	
2	cannabis/	291	
3	(cannabi* or hemp* or marijuana* or nabilone or thc* or sativex or dronabinol or Nabiximol* or epidiolex or Levonantradol or Ajulemic acid or ECP002A or tetrahydrocannabinol).mp.	4554	
4	1 or 2 or 3	4554	
5	exp "Schizophrenia and Disorders with Psychotic Features"/	8104	
6	Tic disorders/	97	
7	tics/	61	
8	Tourette syndrome/	226	
9	exp intellectual disability/	1275	
10	exp Child Development Disorders, Pervasive/	1107	
11	Child behavior disorders/	828	
12	Stress Disorders, Post-Traumatic/	2215	
13	exp anxiety disorders/	7964	
14	exp depression/	10330	
15	exp Depressive Disorder/	10814	
16	exp bipolar disorder/	2399	
17	Fetal Alcohol Spectrum Disorders/	64	
18	(psychosis or psychoses or psychotic or schizophreni* or adhd or attention deficit disorder*).mp.	26645	
19	(tics or tourette* or intellectual development disorder* or intellectual disabilit* or fragile x).mp.	2013	
20	(autism or autistic or Child Behavio?r Disorder* or pervasive development disorder or major depression or f?etal alcohol or post traumatic stress disorder* or posttraumatic stress disorder*).mp.	15983	
21	(anxiety disorder* or severe behavio?ral disturbance* or challenging behavio?r).mp.	9728	
22	exp Epilepsy/	2725	
23	muscle spasticity/	785	
24	exp multiple sclerosis/	2991	
25	chronic pain/	1876	
26	(epilep* or Lennox* or Dravet* or spasticity or cinv or chemotherapy* or chronic pain or multiple sclerosis).mp.	99509	
27	5 or 6 or 7 or 8 or 9 or 10 or 11 or 12 or 13 or 14 or 15 or 16 or 17 or 18 or 19 or 20 or 21 or 22 or 23 or 24 or 25 or 26	165923	
28	4 and 27	1258	
29	adolescent/ or child/ or infant/	130670	
30	28 and 29	147	
31	limit 30 to yr="1980 -Current"	142	

Execute Searches in Ovid

```
Search for: limit 29 to (human and "0300 clinical trial" and (childhood  or adolescence <13 to
17 years>) and yr="1980 -Current")

Results: 1
```

PsycINFO <1806 to September Week 5 2019>			
**#**	**Search Statement**	**Results**	**Annotation**
1	exp cannabinoids/	5283	
2	exp cannabis/	7912	
3	(cannabi* or hemp* or marijuana* or nabilone or thc* or sativex or dronabinol or Nabiximol* or epidiolex or Levonantradol or Ajulemic acid or ECP002A or tetrahydrocannabinol).mp.	25547	
4	1 or 2 or 3	25578	
5	exp psychosis/	113480	
6	exp schizophrenia/	88619	
7	exp attention deficit disorder/	26116	
8	tics/	1724	
9	tourette syndrome/	3082	
10	exp intellectual development disorder/	43900	
11	fragile x syndrome/	1733	
12	exp bipolar disorder/	29584	
13	exp autism spectrum disorders/	41401	
14	exp major depression/	125391	
15	fetal alcohol syndrome/	1715	
16	exp posttraumatic stress disorder/	31398	
17	exp anxiety disorders/	52309	
18	(psychosis or psychoses or psychotic or schizophreni* or adhd or attention deficit disorder*).mp.	213457	
19	(tics or tourette* or intellectual development disorder* or intellectual disabilit* or fragile x).mp.	56470	
20	(autism or autistic or pervasive development disorder or major depression or f?etal alcohol or post traumatic stress disorder* or posttraumatic stress disorder*).mp.	215343	
21	(anxiety disorder* or severe behavio?ral disturbance* or challenging behavio?r).mp.	52396	
22	exp epilepsy/	26722	
23	exp seizures/	15796	
24	multiple sclerosis/	12431	
25	chronic pain/	12873	
26	chemotherapy/	2925	
27	(epilep* or Lennox* or Dravet* or spasticity or cinv or chemotherapy* or chronic pain or multiple sclerosis).mp.	85323	
28	5 or 6 or 7 or 8 or 9 or 10 or 11 or 12 or 13 or 14 or 15 or 16 or 17 or 18 or 19 or 20 or 21 or 22 or 23 or 24 or 25 or 26 or 27	608284	
29	4 and 28	5177	
30	limit 29 to (human and "0300 clinical trial" and (childhood <birth to 12 years> or adolescence <13 to 17 years>) and yr="1980 -Current")	24	

Execute Searches in Ovid
